# Characterization of the Complete Mitochondrial Genome of the Bromeliad Crab *Metopaulias depressus* (Rathbun, 1896) (Crustacea: Decapoda: Brachyura: Sesarmidae)

**DOI:** 10.3390/genes13020299

**Published:** 2022-02-04

**Authors:** Milena A. Rodriguez-Pilco, Peter Leśny, Lars Podsiadłowski, Christoph D. Schubart, Juan Antonio Baeza

**Affiliations:** 1 Facultad de Ciencias Biológicas, Universidad Nacional de San Agustin, Av. Daniel Alcides Carreon s/n, Arequipa 04001, Peru; milealee101@gmail.com; 2 Institute for Evolutionary Biology & Animal Ecology, University Bonn, 53121 Bonn, Germany; peter.lesny@gmx.de; 3 Centre for Molecular Biodiversity Research (ZMB), Zoologisches Forschungsmuseum Alexander Koenig (ZFMK), 53113 Bonn, Germany; lars@cgae.de; 4 Zoology & Evolution, University of Regensburg, 93040 Regensburg, Germany; Christoph.Schubart@biologie.uni-regensburg.de; 5 Department of Biological Sciences, Clemson University, Clemson, SC 29634, USA; 6 Smithsonian Marine Station at Fort Pierce, 701 Seaway Drive, Fort Pierce, FL 34949, USA; 7 Departamento de Biología Marina, Facultad de Ciencias del Mar, Universidad Católica del Norte, Larrondo, Coquimbo 1281, Chile

**Keywords:** mitochondrial DNA, Sanger sequencing, mitogenome, selective pressure

## Abstract

*Metopaulias depressus* is a non-marine crab endemic to Jamaica that dwells in rainforest bromeliads and exhibits elaborate active parental care behavior. Current genomic resources on *M. depressus* are rare, limiting the understanding of its adaptation to terrestrial life in species that evolved from marine ancestors. This study reports the complete mitochondrial genome of *M. depressus* assembled using Sanger sequencing. The AT-rich mitochondrial genome of *M. depressus* is 15,765 bp in length and comprises 13 protein-coding genes (PCGs), 2 ribosomal RNA genes, and 22 transfer RNA genes. A single 691 bp-long intergenic space is assumed to be the control region (CR) or D-loop. A set of selective pressure analyses indicate that the entirety of the PCGs experience purifying selection. *Cox1*, *cox2*, *nad5*, *cox3*, and *atp6* experience strong purifying selection, and *atp8* experiences weak purifying selection compared to the rest of the PCGs. The secondary structures of most tRNA genes exhibit a standard ‘cloverleaf’ structure, with the exception of *trnS1*, which lacks the dihydroxyuridine (DHU) arm but not the loop, the *trnH* gene, which lacks the thymine pseudouracil cytosine (T) loop but not the arm, and *trnM*, which exhibits an overly developed T loop. A maximum likelihood phylogenetic analysis based on all PCGs indicated that *M. depressus* is more closely related to the genera *Clistocoeloma*, *Nanosesarma*, and *Parasesarma* than to *Chiromantes*, *Geosesarma*, and *Orisarma*. This study contributes to deciphering the phylogenetic relationships within the family Sesarmidae and represents a new genomic resource for this iconic crab species.

## 1. Introduction

Within the Decapoda, crabs belonging to the Infraorder Brachyura are recognized for their astonishing anatomical, ecological, physiological, and behavioral diversity [1,2]. Among them, the family Sesarmidae is a speciose clade that has successfully colonized marine intertidal and supratidal zones [3,4,5]. Some lineages have even radiated into freshwater and terrestrial habitats, and these non-marine sesarmids often exhibit abbreviated larval development and complex parental–offspring interactions [6]. *Metopaulias depressus* sets itself apart even within this remarkable family due to its unique forest-dwelling lifestyle and active parental care behavior [3,7,8].

*Metopaulias depressus* is endemic to Jamaica and inhabits epiphytic and bottom-dwelling large bromeliads in central and western rainforests of the island [7]. As in most decapod crabs, embryos of *M*. *depressus* hatch as planktonic larvae, but larval development is abbreviated (9–10 days). Breeding females release their larvae in small water reservoirs located in bromeliad leaf axils, tending their offspring for about eight weeks. Females actively clean the leaf axil of all litter and organic debris except for land snail shells, which are retained. This manipulation in the “nursery axil” improves the dissolved oxygen and the carbon dioxide balance of the axil water and changes the pH from acid to neutral [9,10]. Additionally, female protective behavior reduces the mortality of offspring in the nursery by predatory damselfly nymphs and spiders [11]. Mothers feed their young with prey (i.e., snails, millipedes) caught nearby and carried into the nursery axil [7]. Parental care in this species results in the establishment of a family or a helper group–i.e., a mother and its offspring. This example shows the behavioral plasticity of primarily marine crustaceans when inhabiting unusually harsh, i.e., non-marine, environments.

Active parental care of post-hatching offspring has been observed in other sesarmid crabs that have adapted to adverse terrestrial or semi-terrestrial environments (e.g., *Geosesarma notophorum* [12], *Sesarma jarvisi* [13]). In addition, post-hatching parental care is widely recognized among freshwater Astacidea (e.g., *Procambarus clarkii* [14,15,16]; *Orconectes inermis inermis* and *O. pellucidus* [17]; see review in [18]). These examples show the behavioral plasticity and the potential for advanced social behavior in crustaceans evolving from marine ancestors when colonizing unusually harsh environments.

Despite the remarkable lifestyle and behavior of *M. depressus* and other semi-terrestrial sesarmid crabs, only a limited number of genomic resources exist for these crabs [19,20]), which, in turn, limits the understanding of adaptations to terrestrial life and the genomic mechanisms driving abbreviated development and active parental care. This study forms part of a broader effort aimed at developing genomic resources for comparing marine, semi-terrestrial, and terrestrial crabs, especially those belonging to the subsection Thoracotremata, as it includes most crabs with terrestrial adaptations. This subsection, however, is in need of taxonomic stability, because the most commonly used superfamily classification [21] does not correspond to current knowledge of phylogenetic relationships [22,23,24,25]. Herein, we sequenced and characterized in detail the complete mitochondrial genome of an additional representative of the family Sesarmidae, *M. depressus*, known as one of the most successful and ecologically specialized crabs that became independent of the sea in a relatively short time frame [8]. The comparison with other known thoracotreme mitogenomes will also help to outline and support the establishment of phylogeny-based groupings.

## 2. Materials and Methods

### 2.1. Specimen Collection and Mitochondrial Genome Sequencing

The used specimen was collected during a field trip and visit to the Windor Great House near Sherwood (Trelawny) in Cockpit Country, Jamaica. Collecting permits were obtained beforehand. DNA extraction was conducted using the DNeasy Tissue Kit (Qiagen, Hilden, Germany), following the manufacturer’s protocol. Next, the mitochondrial genome of *M. depressus* was assembled using a primer-walking strategy with the set of primer pairs developed by [26]. More specifically, the whole mitochondrial genome of *M. depressus* was first amplified in three long overlapping PCR products. Next, these products were used as templates for amplifying shorter fragments (PCR products > 800 bp) using the Sanger sequencing method, employing a primer-walking strategy. For more details such as primer sequences and PCR conditions, see [26].

### 2.2. Mitochondrial Genome Annotation and Characterization

The *in silico* annotation of the mitochondrial genome of *M. depressus* was conducted with the web servers MITOS (http://mitos.bioinf.uni-leipzig.de/index.py, accessed on 15 May 2021 [27]) and MITOS 2 (http://mitos2.bioinf.uni-leipzig.de/index.py, accessed on 15 May 2021 [28]) using the invertebrate genetic code. Manual curation of the *in silico* annotation, including start and stop codon corrections, was conducted using the web server Expasy Translate Tool (https://web.expasy.org/translate/, accessed on 15 May 2021 [29]) and the software MEGA 7 [30]. Mitochondrial genome circular visualization was performed with the web server GenomeVx (http://wolfe.ucd.ie/GenomeVx/, accessed on 15 May 2021 [31]).

The nucleotide composition of the entire mitochondrial chromosome and each protein coding gene (PCG) was estimated with the software MEGA 7 [30].

Codon usage of each PCG was estimated using the invertebrate genetic code in the Sequence Manipulation Suite: Codon usage web server (https://www.bioinformatics.org/sms2/codon_usage.html, accessed on 15 May 2021 [32]), and visualization of the Relative Synonymous Codon Usage (RSCU) was performed using the EZcodon tool in the EZmito web server (http://ezmito.unisi.it/ezcodon, accessed on 15 May 2021 [33]).

To explore selective pressures on each mitochondrial PCG, a pairwise comparison was performed between *M. depressus* and *Clistocoeloma sinense* (GenBank: NC_033866). The number of nonsynonymous substitutions per nonsynonymous site (Ka), synonymous substitutions per synonymous site (Ks), and the ratio Ka/Ks (ω) were estimated using the software KaKs_calculator 2.0 [6]. If a PCG experiences neutral selection, then ω = 1. Negative or purifying selection is indicated by values ω < 1, whereas positive or diversifying selection is denoted by values ω > 1. The γ-MYN model was used to account for variable mutation rates along each sequence during calculations [34].

tRNA and their secondary structures were predicted using the program MiTFi [35], as implemented in MITOS and MITOS2. The visualization of the secondary structure for each tRNA was conducted using the FORNA web server (http://rna.tbi.univie.ac.at/forna/, accessed on 15 May 2021 [35,36]).

The control region was examined in detail. First, microsatellites were detected using the web server Microsatellite Repeats Finder (http://insilico.ehu.es/mini_tools/microsatellites/, accessed on 15 May 2021 [37]). Next, the presence of tandem repeats in this region was explored using the web server Tandem Repeats Finder (https://tandem.bu.edu/trf/trf.html, accessed on 15 May 2021 [38]). Lastly, the secondary structure, including the presence of hairpin structures, in the control region was explored using the RNAstructure Secondary Structure web server (https://rna.urmc.rochester.edu/RNAstructureWeb/Servers/Predict1/Predict1.html, accessed on 15 May 2021 [39]).

### 2.3. Phylogenetic Position of Metopaulias Depressus

The phylogenetic position of *M. depressus* among other representatives of the family Sesarmidae was examined based on PCGs. Our analysis was conducted with amino acids instead of nucleotides due to the fact that the phylogenetic signal from nucleotide characters alone has the potential to be saturated. The newly sequenced and annotated mitogenome of *M. depressus*, together with those of 11 other species (6 genera) belonging to the family Sesarmidae available in GenBank (consulted: 19 December 2021) were used for the phylogenetic analysis conducted using the software MitoPhAST V2.0 [40].

Outgroups included species from each of the families Grapsidae, Gecarcinidae, Ocypodidae, Xenograpsidae, and Varunidae. MitoPhAST first extracted all 13 PCG nucleotide sequences from the species available in GenBank and any others provided by the user (i.e., *M. depressus*). Next, each PCG nucleotide sequence was translated to amino acids and each PCG amino acid sequence was then aligned using Clustal Omega [41,42]. Poorly aligned regions were removed with trimAl v1.2.0 [43] before the dataset was partitioned and the best fitting models of sequence evolution were selected with ProtTest3 v3.4 [44]. Lastly, the concatenated and partitioned PCG amino acid alignments were used to perform a maximum likelihood phylogenetic tree search in the software IQ-TREE [45]. The robustness of the ML tree topology was ascertained by 1000 bootstrap pseudoreplicates of the tree search.

## 3. Results and Discussion

**Figure 1:**
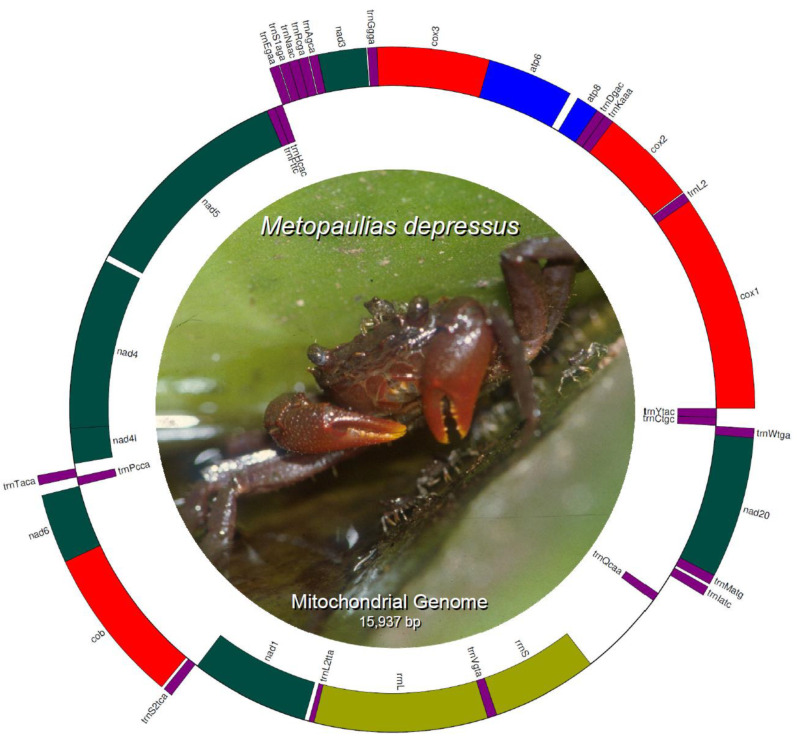
Circular genome map of *Metopaulias depressus* mitochondrial DNA. Photo credit: Rudolph Diesel.

The mitochondrial genome of *Metopaulias depressus* (KX118277) is 15,765 bp in length and encodes 13 protein coding genes (PCGs), 22 transfer RNA genes, 2 ribosomal RNA genes (rrnL [16S] and rrnS [12S]), and a single, relatively long (691 bp) non-coding putative control region. Most of the PCGs and tRNA genes are encoded on the L-strand, whereas only four PCGs (*nad5*, *nad4*, *nad4l*, and *nad1*), the two ribosomal RNA genes, and eight tRNA genes (trnH, trnF, trnP, trnL2, trnQ, trnV, trnC, and trnY) are encoded in the H-strand (Table 1) (Figure 1). Gene order and strand arrangement in *M. depressus* is identical to that reported before in all co-familiar species (except *G. penangense* [46]) with mitochondrial genomes deposited in GenBank (i.e., *O. neglectum, O. sinense, P. bidens,* and *P. tripectinis*, among others [47,48,49,50]). In contrast to the gene arrangement observed in all sesarmid crabs, with a *trnQ*-*trnI*-*trnM*, *G. penangense* exhibits a *trnI*-*trnQ*-*trnM* gene arrangement [46]. Compared to other decapod infraorders, brachyuran crabs (infraorder Brachyura) contain a translocation of the *trnH* gene between the *trnE* and *trnF* genes, rather than between the *nad5* and *nad4* genes [5]. This translocation is present in *M. depressus* and all co-familiar species [5,47,48,49,51].

The overall nucleotide composition of the mitochondrial genome’s light DNA strand was as follows: A = 37.9%, G = 8.7%, C = 14%, and T = 39.4%, with a GC-content equal to 22.7% and an AT-content equal to 77.3%. This nucleotide usage is within the range reported for other sesarmid crab species (Supplementary Table S1). The highest AT-content value has been reported for *Geosesarma penangense* (78.44%) [46], whereas the lowest reported AT-content value belongs to *Parasesarma tripectinis* (74.22%) [50]. AT-skewed mitochondrial genomes are often reported across metazoan clades, including crustaceans and brachyuran crabs [48,52,53].

In the mitochondrial genome of *M. depressus*, PCGs comprise a total of 3673 codons. Seven (*cox1*, *cox2*, *atp8*, *cox3*, *nad4*, *cob*, and *nad2*) and five (*nad3*, *nad5*, *nad6*, *nad1*, and *nad4l*) of the 13 PCGs use ATG and ATA, respectively, as start codon, whereas *atp6* uses ATT as start codon. Nine PCGs use TAA (*cox2*, *atp8*, *atp6*, *cox3*, *nad5*, *nad4*, *nad4l*, *nad6*, and *nad1*) as stop codon and two PCGs use TAG (*cox1* and *nad2*). Lastly, two genes (*nad3* and *cob*) exhibit incomplete (T) stop codons (Table 1). An incomplete stop codon in the *cob* gene is also observed in the co-familiar species *Parasesarma affine, P. pictum, O. neglectum*, and *E. lafondii* [5,6,47,51]. It is assumed that truncated stop codons are completed via post-transcriptional polyadenylation ([54] and references therein).

**Figure 2:**
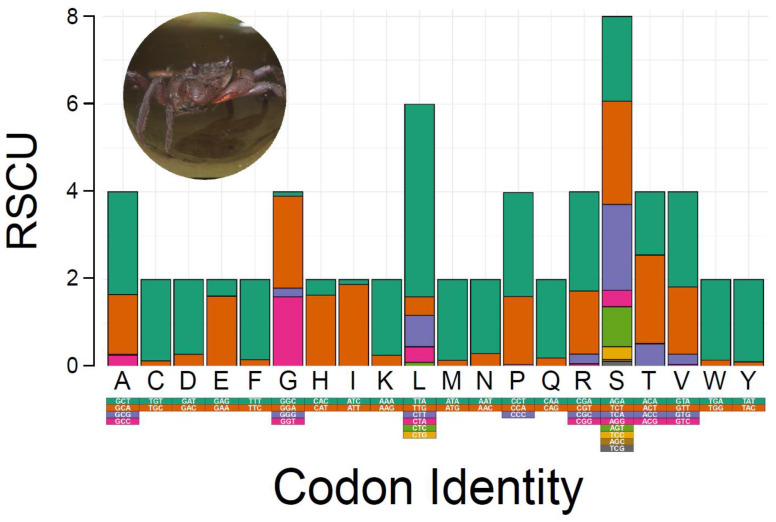
Relative synonymous codon usage (RSCU) in *Metopaulias depressus.* Photo credit: Rudolph Diesel.

Relative synonymous codon usage (RSCU) and amino acid composition in the PCGs of *M. depressus* are summarized in Figure 2. The most frequently used codons (amino acids) were: TTA (Leu) used 434 times (73%), ATT (Ile) used 336 times (94%), TTT (Phe) used 317 times (91%), and ATA (Met) used 225 times (92%). Codons (amino acids) that were the least commonly used to encode their respective amino acids (excluding stop codons) included CGC (Ala), used one time (0.01%), CTG (Leu), used one time (undefined %), CGG (Arg), used one time (0.02%), AGC (Ser), used two times (0.01%), and CCC (Pro,) used two times (0.02%) (Supplementary Table S2). RSCU and amino acid composition of PCGs in *M. depressus* is similar to that reported before in other representatives of the family Sesarmidae. For instance, the most frequently used codons in *P. affine*, *O. sinense*, and *P. bidens* were Leu, Ile, and Phe, in agreement with that observed in this study for *M. depressus* [5,48,49]. In addition to *M. depressus*, codons for Met are frequently used in *P. pictum* [6]. All the codons coding for the aforementioned amino acids are AT-rich, in line with the observed overrepresentation of A and T nucleotides in the mitogenome of *M. depressus* and other co-familiar crabs [5,46].

**Figure 3:**
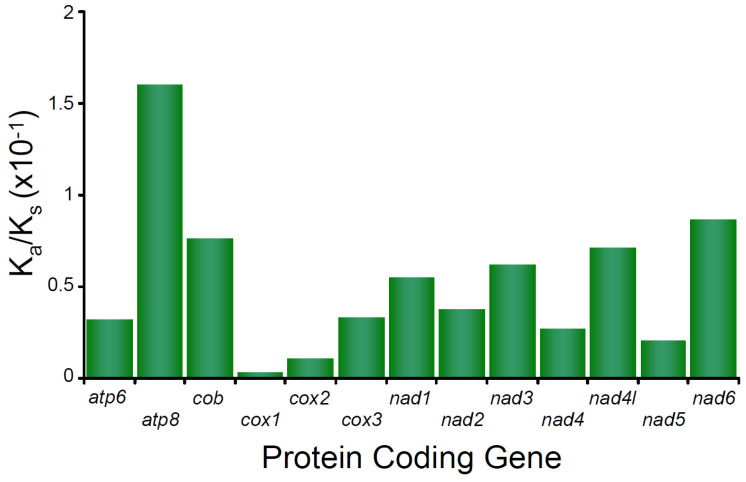
Selective pressure analysis in the protein coding genes of *Metopaulias depressus*.

In the mitochondrial genome of *M. depressus*, the Ka/Ks ratio estimated for all PCGs show values < 1 PCGs (all *p* values < 0.05), indicating that purifying selection is acting upon all these PCGs. The Ka/Ks ratio estimated for *atp8* is the highest observed value (0.16017) compared to the rest of the PCGs and indicates that the purifying selection was relatively weak in this gene. In turn, Ka/Ks ratios calculated for *cox1*, *cox2*, *nad5*, *cox3*, and *atp6* are the lowest observed values (0.029, 0.01069, 0.02027, 0.03302, and 0.03196, respectively) and indicate strong selective pressure affecting the latter PCGs (Figure 3). Selective pressure in PCGs has not been studied before in any other crab belonging to the family Sesarmidae. However, a strong pattern of purifying selection has been reported for many other brachyuran crabs, crustaceans, and arthropods in general ([34] and references therein). A recent study of caridean shrimps (genus *Synalpheus*) found a relationship between PCG length and the strength of purifying selection, with short genes (e.g., *atp8*) being subject to weaker purifying selection than longer PCGs [55]. Our observations are in agreement with the aforementioned pattern. Whether or not an association between gene length and the strength of purifying selection exists in sesarmid and other brachyuran crabs remains to be addressed.

**Figure 4:**
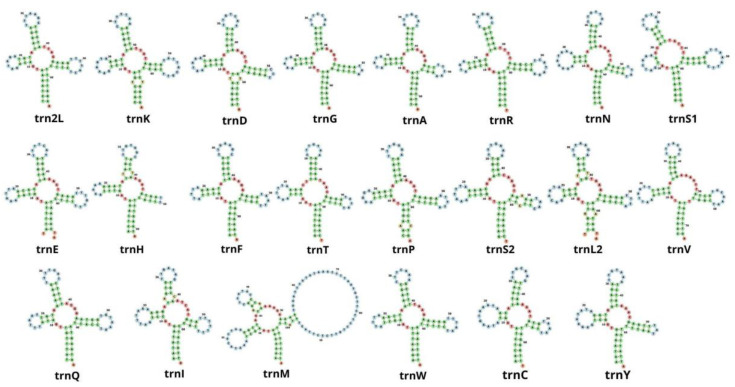
Secondary structures of 22 transfer RNA genes in *Metopaulias depressus*.

In the mitochondrial genome of *M. depressus,* 19 out of the 22 tRNA genes exhibited a cloverleaf secondary structure (Figure 4). The *trnS1* gene exhibited a deletion of the dihydroxyuridine (DHU) arm, having only its loop. Other co-familiar crabs, including *O. sinense*, *P. pictum*, *P. affine*, *P. bidens*, *G. faustum*, *G. penangense*, *C. sinense*, and *C. haematocheir*, presented the same deletion of the DHU arm in the *trnS1* gene [5,6,46,48,49,51,52,53,54,55,56,57,58], with the exception of *O. neglectum* [47], in which all tRNAs exhibited the typical cloverleaf secondary structure. A truncated *trnS1* gene represents a conserved mitochondrial feature in eumetazoans, including crabs and other decapod crustaceans [6,49,56].

Unexpectedly, we found two other tRNA genes with a secondary structure that deviates from the expected ‘cloverleaf’ shape: the *trnH* gene lacks the thymine pseudouracil cytosine (T) loop, and the *trnM* exhibits an overly developed T loop (Supplementary Figure S1). Some studies have examined the secondary structure of mitochondrial tRNA genes in co-familiar species (*Orisarma sinense* as *C. haematocheir* [57], *O. sinense* [48], *P. pictum* [6], *P. affine* [5], *P. bidens* [49], *E. lafondii* [51], *G. penangense* and *G. faustum* [46], *O. neglectum* [47], and *C. sinense* [58]), and truncated arms have also been observed in *G. penangense* (*trnC*), *G. faustum* (*trnD*, *trnH*, and *trnR*) [46], and *C. haematocheir* (*trnS*) [57]. Whether or not truncated tRNAs are functional remains to be explored. It has been hypothesized that tRNA editing after the translation of truncated tRNAs might make them functional [59]. 

In *M. depressus*, the 691 bp-long control region (CR) is located between the rrnS and *trnQ* genes, starting at position 13,480 and ending at position 14,170. The length of the CR was similar in range (630 to 751 bp) to that previously reported in other crabs belonging to the family Sesarmidae [5,6,47,48,49,58]. The Microsatellite Repeats Finder analysis found 18 TA-rich microsatellites (SSRs) distributed from position 57 to 680. Most SSRs exhibited TA, AA, and TT di-nucleotide repeats (Supplementary Table S3). The tandem repeat analysis identified one TA-rich tandem repeat, 17 bp in length, repeated four times and located between positions 572 and 637 of the CR. The RNA structure Web Server tool revealed 20 possible secondary structures (Gibbs free energy (ΔG) ranged from −77.8 to −76.7 kcal/mol, Supplementary Figure S2), and in all of them, hairpin structures were observed along most of the entire length of this region. A detailed characterization of the CR is not available for any other sesarmid crab. However, the presence of SSRs, tandem repeats, and numerous hairpin secondary structures are often observed in the CR of other brachyuran crabs as well as other closely or more distantly related decapod crustaceans (e.g., [34] and references therein).

**Figure 5:**
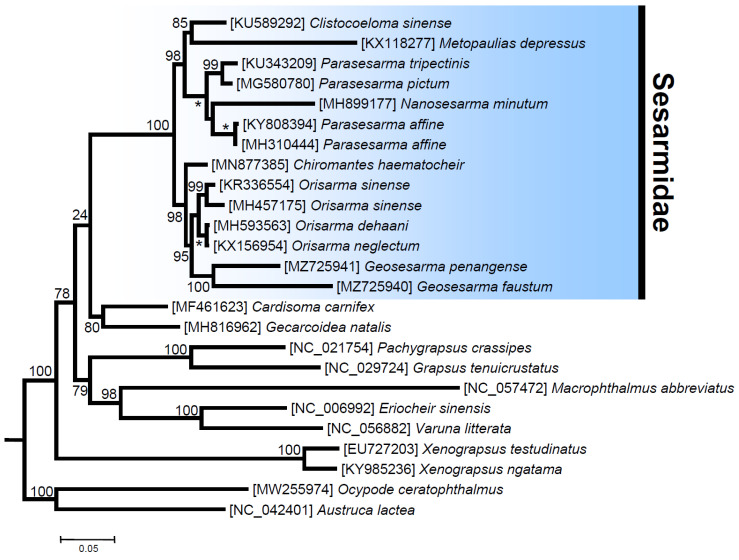
Total evidence phylogenetic tree obtained from ML analysis based on a concatenated alignment of amino acids of the 13 protein-coding genes present in the mitochondrial genome of *Metopaulias depressus* and other representatives of the family Sesarmidae. Outgroups included a total of four species belonging to the families Gecarcinidae and Xenograpsidae. The robustness of the ML tree topology was ascertained by 1000 bootstrap pseudoreplicates (numbers above or below the nodes) of the tree search. *: full support, boostrap value = 100.

The ML phylogenetic tree with various representatives of the Thoracotremata (25 terminals, 3695 amino acid characters, and 1074 informative sites) fully supports the monophyly of the family Sesarmidae and the other selected crab families (Ocypodidae, Grapsidae, Varunidae, Xenograpsidae, and Gecarcinidae), with bootstrap values (bv) of 100 (except for the Gecarcinidae with bv = 80). Even if inter-familiar relationships are not fully resolved, clear trends become visible. The Ocypodidae, with fiddler and ghost crabs, splits off first, so that all other included families group together as a clear-cut monophylum. This serves as additional evidence that the former superfamily Ocypodoidea has to be redefined with exclusion of the family Macrophthalmidae, for which we provide additional evidence that the latter forms a sister taxon to the Varunidae (bv = 98) (see also [23,24,25]). This will require redefinition of the Grapsoidea at the same time, and one solution is to create a separate superfamily for the Sesarmidae. Within this family, two well-supported clades comprise representatives belonging to the genera *Clistocoeloma* + *Metopaulias* + *Nanosesarma* + *Parasesarma* (CMNPP clade, bv = 98) and *Chiromantes* + *Geosesarma* + *Orisarma* (CCGO clade, bv = 97). In the first CMNPP clade, *Metopaulias* and *Clistocoeloma* form a well-supported clade (bv = 85), sister to representatives of the genera *Parasesarma* and *Nanosesarma* (bv = 100). Within the second clade, the genus *Parasesarma* appears paraphyletic due to the position of *Nanosesarma minutum*, but the latter genus is in need of revision, because it currently includes all small-sized representatives of the family (Figure 5). In the first CCGO clade, the real *Chiromantes haematocheir* (as *Cristarma eulimene* in GenBank, moleculary re-assigned in [60]) is sister to all other species comprised in this clade. The two species of *Geosesarma* used in this analysis cluster together as a fully supported monophyletic clade. With the species re-assignment [60], the monophyly of the genus *Orisarma* becomes well supported, considering that the record of “*Chiromantes haematocheir*” is shown to be another representative of *Orisarma sinense* [60] and the two together are sister to a second clade that comprises *O. dehaani* and *O. neglectum* (Figure 5). Overall, the phylogenetic relationships among genera and families reported in this study are not in full agreement with inferences drawn by previous phylogenetic studies that used complete mitochondrial genomes. However, these included a smaller number of species belonging to the family Sesarmidae and other and fewer members of the Thoracotremata than were included in the present study ([6] and references therein). 

## 4. Conclusions

This study sequenced and characterized in detail the mitochondrial genome of the bromeliad crab *Metopaulias depressus*. Characterization of the complete mitochondrial genome of *M. depressus* enhances the genomic resources available for the family Sesarmidae and the Thoracotremata and Brachyura in general, particularly its radiation into semi-terrestrial and terrestrial environments. Present and future mitochondrial genomes assembled for other species in these taxa will permit the exploration of the interlink between the colonization of harsh, i.e., non-marine, including terrestrial, environments from marine ancestral species and selective pressures and rates of molecular evolution in mitochondrial genomes.

## Figures and Tables

**Table 1:** Mitochondrial genome of *Metopaulias depressus*. Arrangement and annotation.

Name	Type	Start	Stop	Strand	Length (bp)	Start	Stop	Anticodon	Continuity
cox1	pcg	1	1560	+	1560	ATG	TAG		−25
trnL2	tRNA	1536	1601	+	66			TAA	+8
cox2	pcg	1610	2317	+	708	ATG	TAA		−20
trnK(aaa)	tRNA	2298	2366	+	69			TTT	+1
trnD(gac)	tRNA	2368	2433	+	66			GTC	0
atp8	pcg	2434	2592	+	159	ATG	TAA		+59
atp6	pcg	2652	3260	+	609	ATT	TAA		−1
cox3	pcg	3260	4051	+	792	ATG	TAA		0
trnG(gga)	tRNA	4052	4116	+	65			TCC	+9
nad3	pcg	4126	4464	+	340	ATA	T		+1
trnA(gca)	tRNA	4466	4528	+	63			TGC	+6
trnR(cga)	tRNA	4535	4602	+	67			TCG	+2
trnN(aac)	tRNA	4605	4671	+	67			GTT	+2
trnS1(aga)	tRNA	4674	4740	+	67			TCT	+5
trnE(gaa)	tRNA	4746	4813	+	68			TTC	+3
trnH(cac)	tRNA	4817	4878	−	62			GTG	+1
trnF(ttc)	tRNA	4880	4944	−	65			GAA	0
nad5	pcg	4945	6639	−	1695	ATA	TAA		+51
nad4	pcg	6691	8043	−	1353	ATG	TAA		−7
nad4l	pcg	8037	8312	−	276	ATA	TAA		+37
trnT(aca)	tRNA	8350	8416	+	67			TGT	0
trnP(cca)	tRNA	8417	8483	−	67			TGG	+8
nad6	pcg	8492	8980	+	489	ATA	TAA		0
cob	pcg	8980	10,084	+	1105	ATG	T		+21
trnS2(tca)	tRNA	10,106	10,173	+	68			TGA	+19
nad1	pcg	10,193	11,128	−	936	ATA	TAA		+36
trnL2(tta)	tRNA	11,165	11,235	−	71			TAA	−29
rrnL	rib	11,206	12,574	−	1369				0
trnV(gta)	tRNA	12,575	12,647	−	73			TAC	0
rrnS	rib	12,648	13,479	−	832				0
CR		13,480	14,170		691				0
trnQ(caa)	tRNA	14,171	14,238	−	68			TTG	+168
trnI(atc)	tRNA	14,407	14,473	+	67			GAT	+21
trnM(atg)	tRNA	14,495	14,569	+	75			CAT	−6
nad2	pcg	14,564	15,562	+	999	ATG	TAG		−2
trnW(tga)	tRNA	15,561	15,628	+	68			TCA	+5
trnC(tgc)	tRNA	15,634	15,698	−	65			GCA	0
trnY(tac)	tRNA	15,699	15,765	−	67			GTA	0

## Data Availability

The mitochondrial genome has been deposited in the NCBI with accession number KX118277.

## Supplementary Materials

The following are available online at https://www.mdpi.com/article/10.3390/genes13020299/s1, Figure S1: tRNA-M gene secondary structure of *Metopaulias depressus* exhibiting an unusually developed loop in the T arm; Figure S2: Secondary structure prediction of the control region (CR) in the mitochondrial genome of *Metopaulias depressus*. Table S1: Nucleotide usage, AT-content, and GC-content in crabs belonging to the family Sesarmidae; Table S2: Codon usage analysis of protein coding genes (PCGs) in the mitochondrial genome; Table S3: Microsatellites present in the control region (CR) of *Metopaulias depressus*.
