# Supplementary material for: Characterization of the Complete Mitochondrial Genome of the Bromeliad Crab Metopaulias depressus (Rathbun, 1896) (Crustacea: Decapoda: Brachyura: Sesarmidae)
Source: Genes (Basel). 2022 Feb 4;13(2):299. doi: 10.3390/genes13020299 (PMC8872168; doi:10.3390/genes13020299)
Supplement: Supplementary file 1 [file genes-13-00299-s001.zip › genes-1551490-supplementary.pdf]

Characterization of the complete mitochondrial genome of the bromeliad crab *Metopaulias depressus* (Rathbun, 1896) (Decapoda: Brachyura: Sesarmidae)

Milena A. Rodriguez-Pilco 1, Peter Leśny 2, Lars Podsiadłowski 2, Christoph Schubart 3, J. Antonio Baeza 4,5,6

1 Facultad de Ciencias Biológicas, Universidad Nacional de San Agustin, Av. Daniel Alcides Carreon s/n, Arequipa, Perú

2 Centre for Molecular Biodiversity Research (ZMB), Zoologisches Forschungsmuseum Alexander Koenig (ZFMK), Bonn, Germany

3 Zoology & Evolution, University of Regensburg, 93040 Regensburg, Germany

4 Department of Biological Sciences, 132 Long Hall, Clemson University, Clemson, SC 29634, USA

5 Smithsonian Marine Station at Fort Pierce, 701 Seaway Drive, Fort Pierce, FL 34949, USA

6 Departamento de Biología Marina, Facultad de Ciencias del Mar, Universidad Católica del Norte, Larrondo 1281, Coquimbo, Chile

Supplementary Materials

Table S1: Nucleotide usage, AT-content, and GC-content in crabs belonging to the family Sesarmidae.

Species	Size (bp)	A%	G%	T%	C%	A+T%	G+C%
Orisarma dehaani	15917	37.46	9.49	38.23	14.83	75.69	24.31
Christarma eulimene	15894	37.13	9.71	38.40	14.76	75.53	24.47
Chiromantes haematocheir	15899	37.33	9.44	38.28	14.95	75.61	24.39
Clistocoeloma sinense	15706	37.10	9.40	38.60	14.90	75.70	24.30
Geosesarma faustum	15880	38.70	8.30	39.70	13.20	78.40	21.50
Geosesarma penangense	15955	38.30	8.51	40.14	13.06	**78.44**	21.56
Metopaulias depressus	15765	37.94	8.71	39.38	13.97	77.32	22.68
Nanosesarma minutum	15637	37.97	8.91	39.71	13.40	77.69	22.31
Parasesarma affine	15638	36.60	10.07	38.23	15.10	74.83	25.17
Parasesarma pictum	15611	36.60	9.83	39.00	14.57	75.60	24.40
Parasesarma tripectinis	15612	36.23	10.11	37.98	15.67	**74.22**	25.78
Perisesarma bidens	15641	36.61	10.06	38.20	15.13	74.81	25.19
Orisarma neglectum	15920	37.42	9.51	38.21	14.86	75.63	24.37
Orisarma sinense	15905	37.37	9.39	38.31	14.93	75.68	24.32

Table S2: Codon usage analysis of protein coding genes (PCGs) in the mitochondrial genome of *Metopaulias depressus.*

Amino Acid	Codon	Number	/1000	Fraction
Ala	GCG	1	0.27	0.01
	GCA	55	14.97	0.34
	GCT	95	25.86	0.59
	GCC	11	2.99	0.07
Cys	TGT	26	7.08	0.93
	TGC	2	0.54	0.07
Asp	GAT	52	14.16	0.85
	GAC	9	2.45	0.15
Glu	GAG	15	4.08	0.19
	GAA	64	17.42	0.81
Phe	TTT	317	86.31	0.91
	TTC	30	8.17	0.09
Gly	GGG	10	2.72	0.05
	GGA	107	29.13	0.52
	GGT	82	22.33	0.4
	GGC	5	1.36	0.02
His	CAT	64	17.42	0.82
	CAC	14	3.81	0.18
Ile	ATT	336	91.48	0.94
	ATC	20	5.45	0.06
Lys	AAG	14	3.81	0.13
	AAA	90	24.5	0.87
Leu	TTG	42	11.43	0.07
	TTA	434	118.16	0.73
	CTG	1	0.27	0
	CTA	35	9.53	0.06
	CTT	71	19.33	0.12

	CTC	10	2.72	0.02
Met	ATG	20	5.45	0.08
	ATA	225	61.26	0.92
Asn	AAT	133	36.21	0.84
	AAC	25	6.81	0.16
Pro	CCG	0	0	0
	CCA	49	13.34	0.39
	CCT	75	20.42	0.6
	CCC	2	0.54	0.02
Gln	CAG	8	2.18	0.11
	CAA	68	18.51	0.89
Arg	CGG	1	0.27	0.02
	CGA	30	8.17	0.57
	CGT	19	5.17	0.36
	CGC	3	0.82	0.06
Ser	AGG	17	4.63	0.05
	AGA	87	23.69	0.24
	AGT	41	11.16	0.11
	AGC	2	0.54	0.01
	TCG	6	1.63	0.02
	TCA	88	23.96	0.24
	TCT	106	28.86	0.29
	TCC	13	3.54	0.04
Thr	ACG	1	0.27	0.01
	ACA	62	16.88	0.36
	ACT	87	23.69	0.51
	ACC	22	5.99	0.13
Val	GTG	12	3.27	0.06
	GTA	108	29.4	0.54
	GTT	76	20.69	0.38
	GTC	3	0.82	0.02

Trp	TGG	8	2.18	0.08
	TGA	91	24.78	0.92
Tyr	TAT	152	41.38	0.94
	TAC	10	2.72	0.06
End	TAG	2	0.54	0.18
	TAA	9	2.45	0.82

Table S3: Microsatellites present in the control region (CR) of *Metopaulias depressus*.

Position	Cicle	Repeats	Sequence
57	2	3	AAAAAA
72	3	3	TATTATTAT
97	2	5	ATATATATAT
109	2	3	ATATAT
204	2	5	AAAAAAAAAA
216	2	3	ATATAT
283	2	3	TATATA
334	2	3	ATATAT
350	2	3	TATATA
397	2	3	ATATAT
513	2	3	TATATA
571	2	4	TATATATA
581	2	3	TATATA
598	2	4	ATATATAT
608	2	3	ATATAT
614	4	4	TATATATATATATAT A
665	3	3	TATTATTAT
680	2	3	TTTTTT

Fig S1: tRNA-M gene secondary structure of *Metopaulias depressus* exhibiting an unusually developed loop in the T arm.

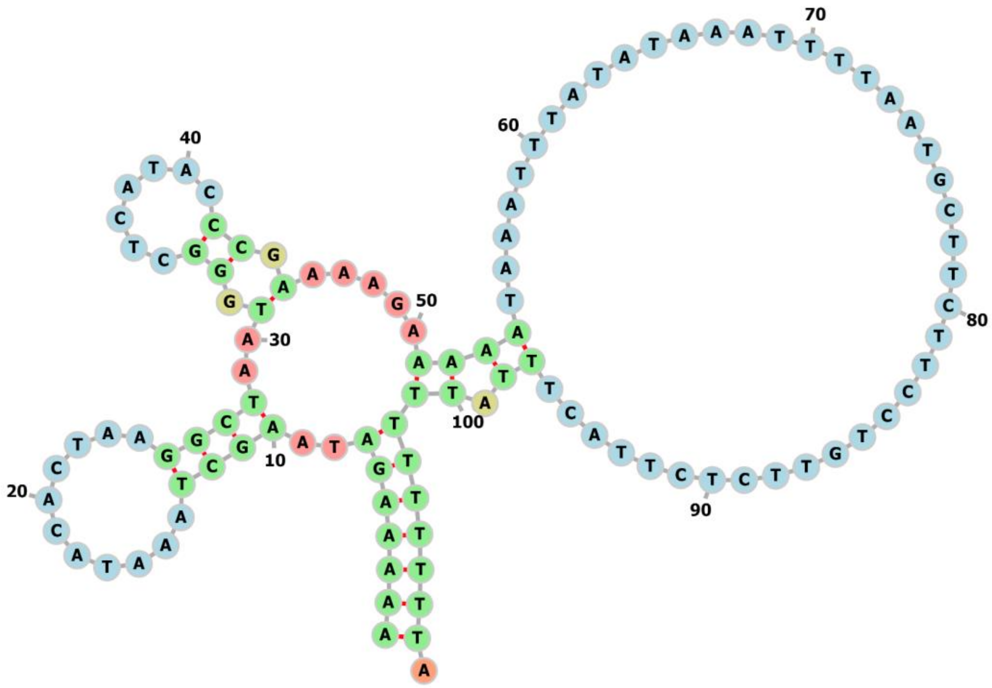

Fig S2: Secondary structure prediction of the control region (CR) in the mitochondrial genome of *Metopaulias depressus*

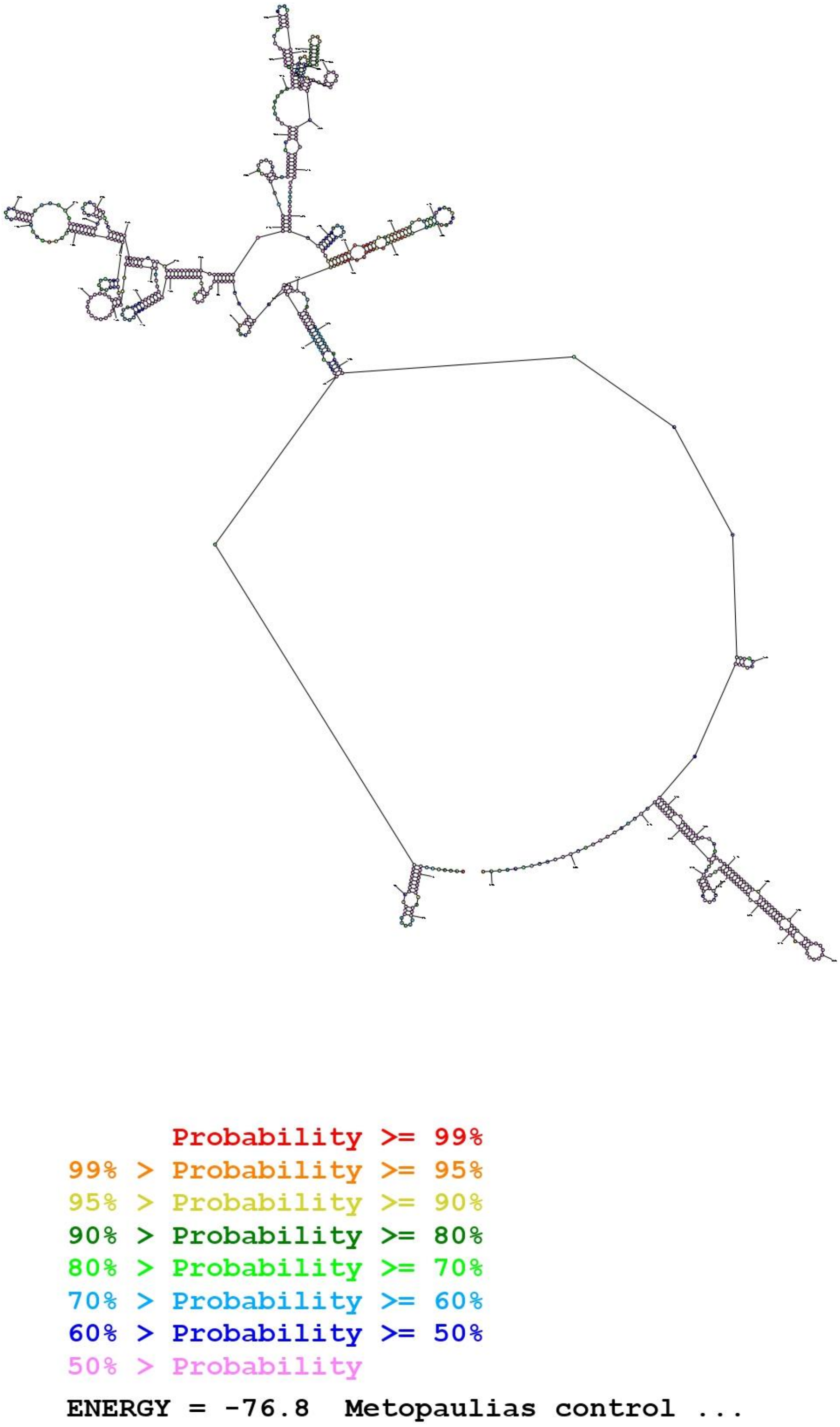

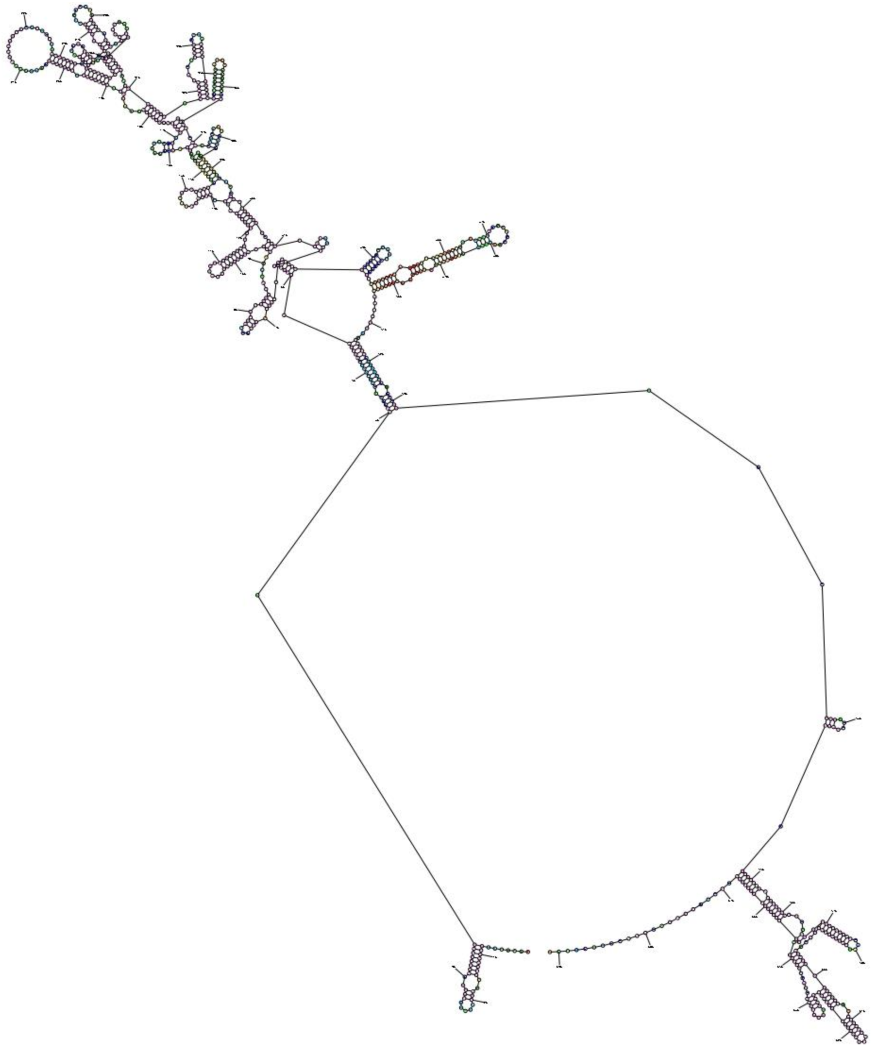

Probability >= 99%
99% > Probability >= 95%
95% > Probability >= 90%
90% > Probability >= 80%
80% > Probability >= 70%
70% > Probability >= 60%
60% > Probability >= 50%
50% > Probability

ENERGY = -76.8 Metopaulias control ...

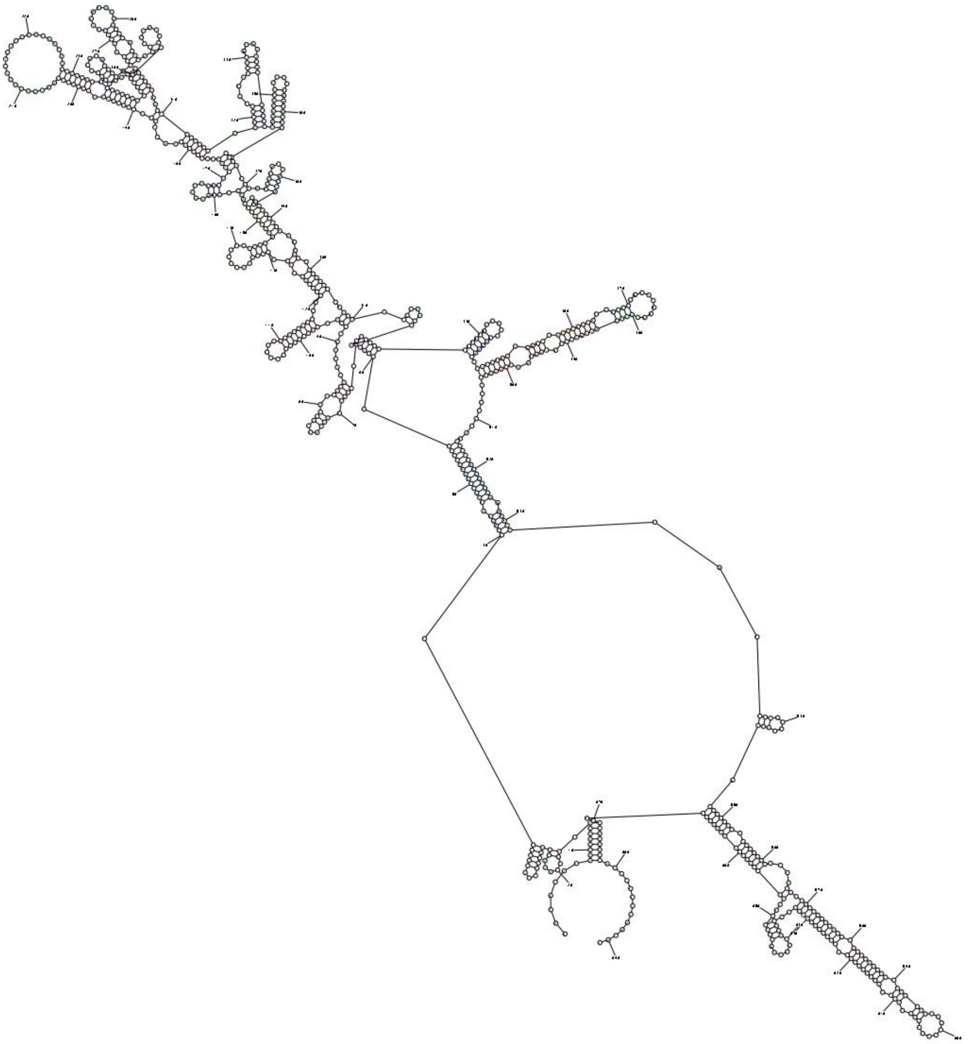

Probability >= 99%
99% > Probability >= 95%
95% > Probability >= 90%
90% > Probability >= 80%
80% > Probability >= 70%
70% > Probability >= 60%
60% > Probability >= 50%
50% > Probability

ENERGY = -76.7 Metopaulias control ...

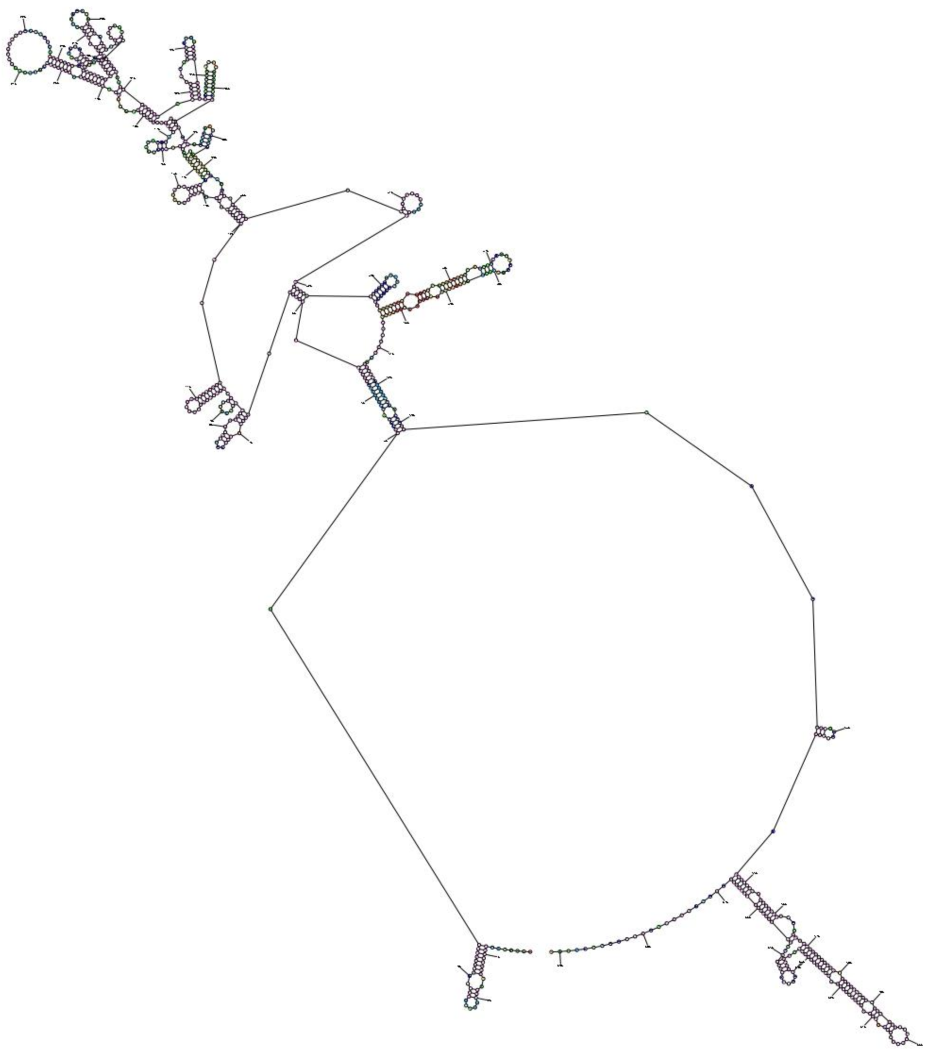

Probability >= 99%
99% > Probability >= 95%
95% > Probability >= 90%
90% > Probability >= 80%
80% > Probability >= 70%
70% > Probability >= 60%
60% > Probability >= 50%
50% > Probability

ENERGY = -76.7 Metopaulias control ...

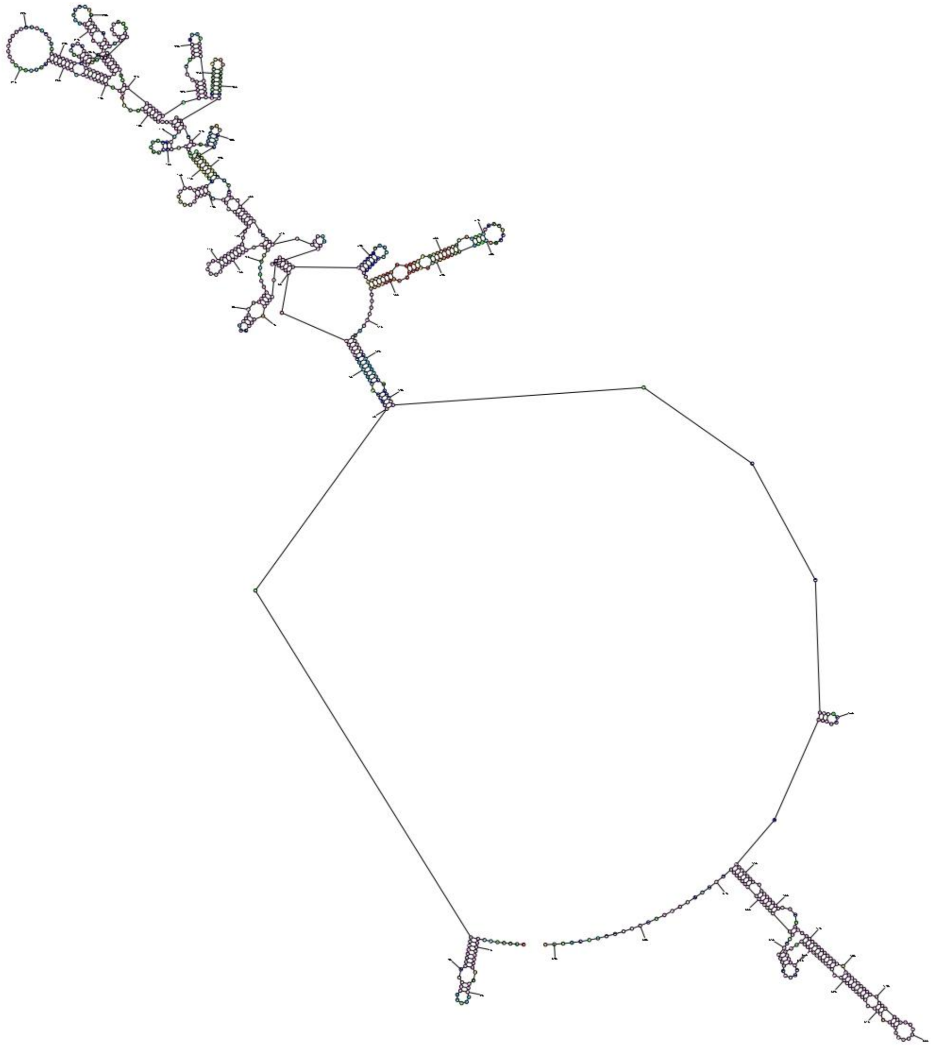

Probability >= 99%
99% > Probability >= 95%
95% > Probability >= 90%
90% > Probability >= 80%
80% > Probability >= 70%
70% > Probability >= 60%
60% > Probability >= 50%
50% > Probability

ENERGY = -77.8 Metopaulias control ...

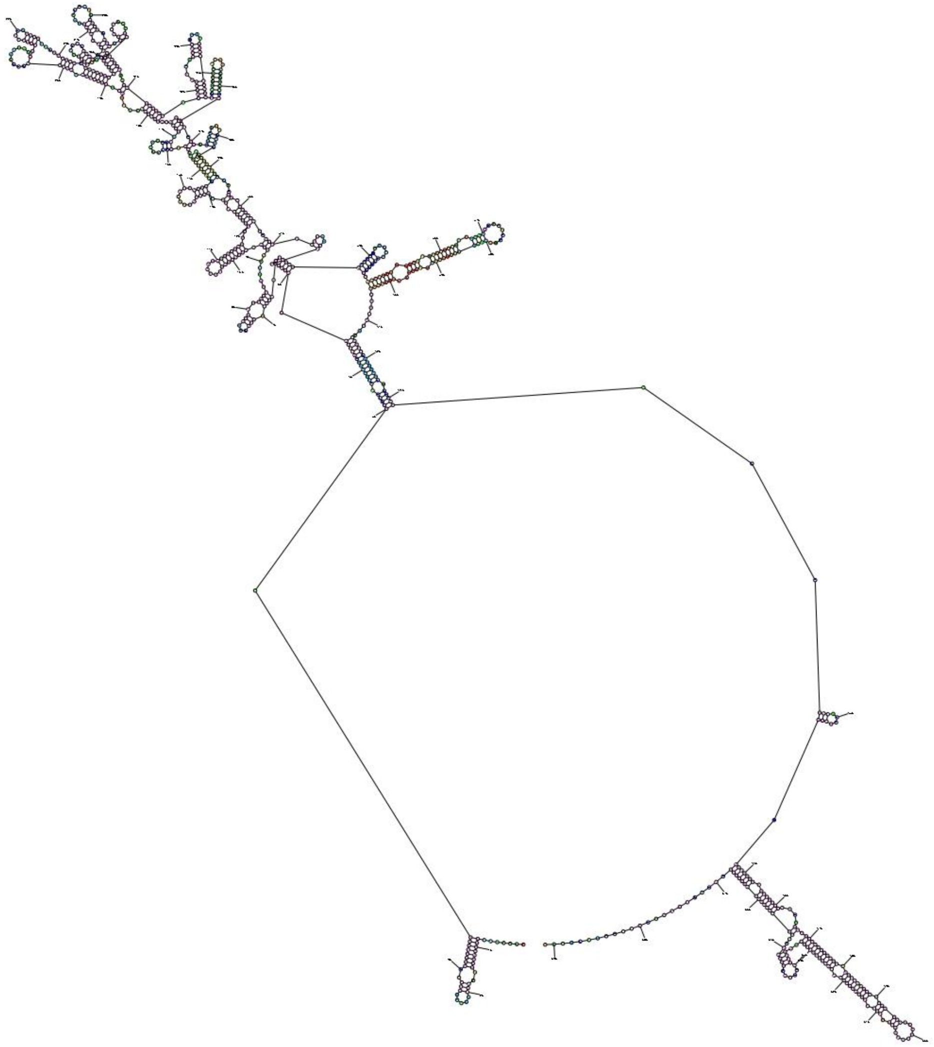

Probability >= 99%
99% > Probability >= 95%
95% > Probability >= 90%
90% > Probability >= 80%
80% > Probability >= 70%
70% > Probability >= 60%
60% > Probability >= 50%
50% > Probability

ENERGY = -77.8 Metopaulias control ...

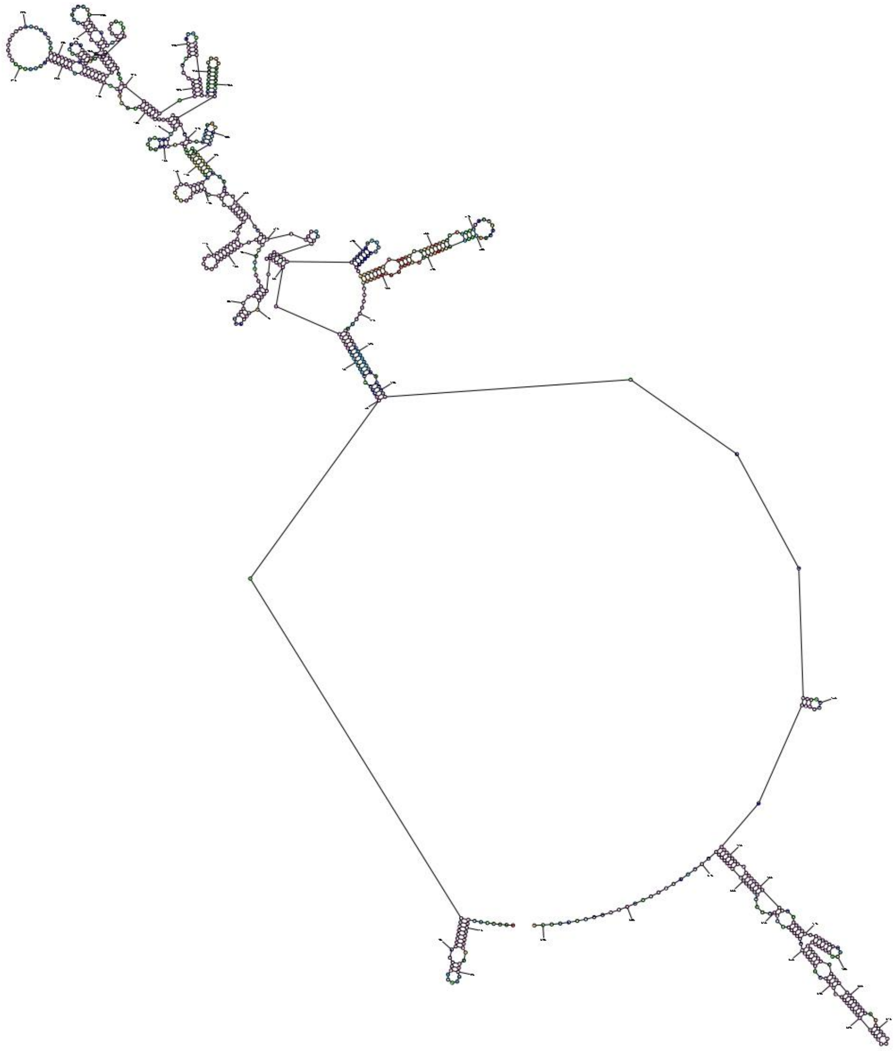

Probability >= 99%
99% > Probability >= 95%
95% > Probability >= 90%
90% > Probability >= 80%
80% > Probability >= 70%
70% > Probability >= 60%
60% > Probability >= 50%
50% > Probability

ENERGY = -77.7 Metopaulias control ...

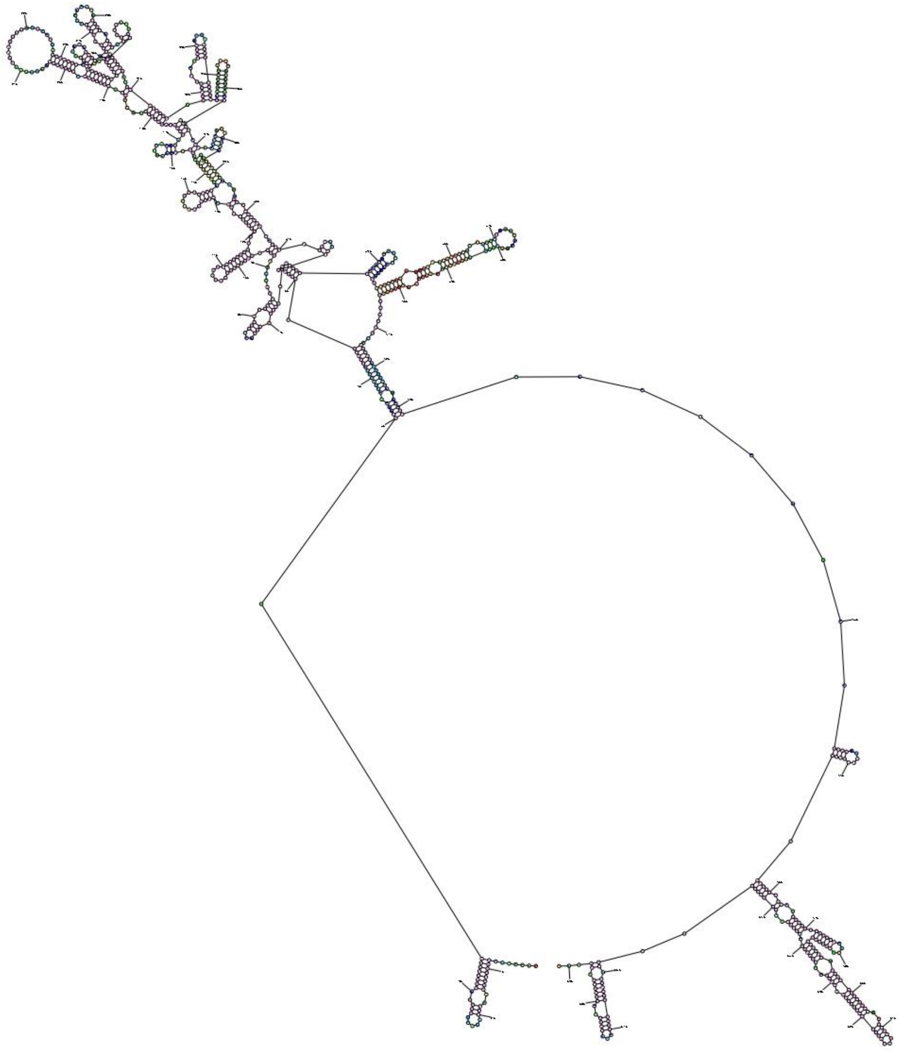

Probability >= 99%
99% > Probability >= 95%
95% > Probability >= 90%
90% > Probability >= 80%
80% > Probability >= 70%
70% > Probability >= 60%
60% > Probability >= 50%
50% > Probability

ENERGY = -77.6 Metopaulias control ...

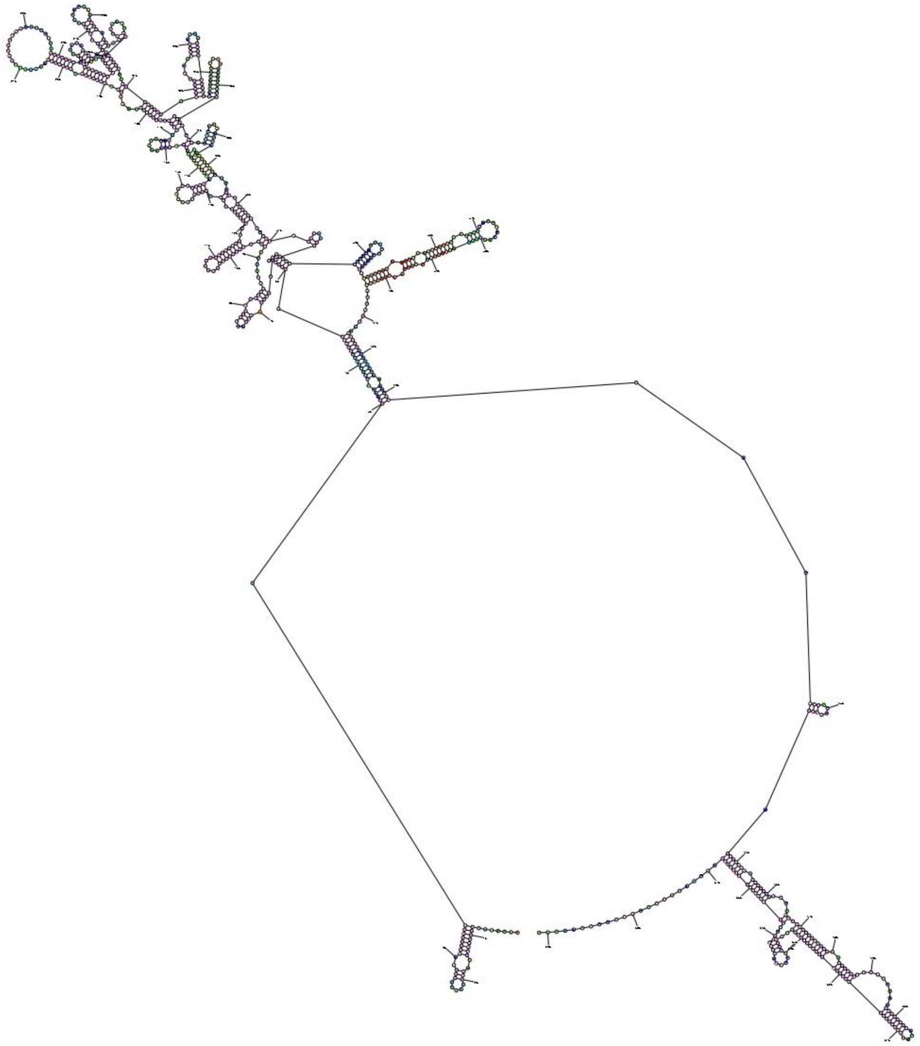

Probability >= 99%
99% > Probability >= 95%
95% > Probability >= 90%
90% > Probability >= 80%
80% > Probability >= 70%
70% > Probability >= 60%
60% > Probability >= 50%
50% > Probability

ENERGY = -77.6 Metopaulias control ...

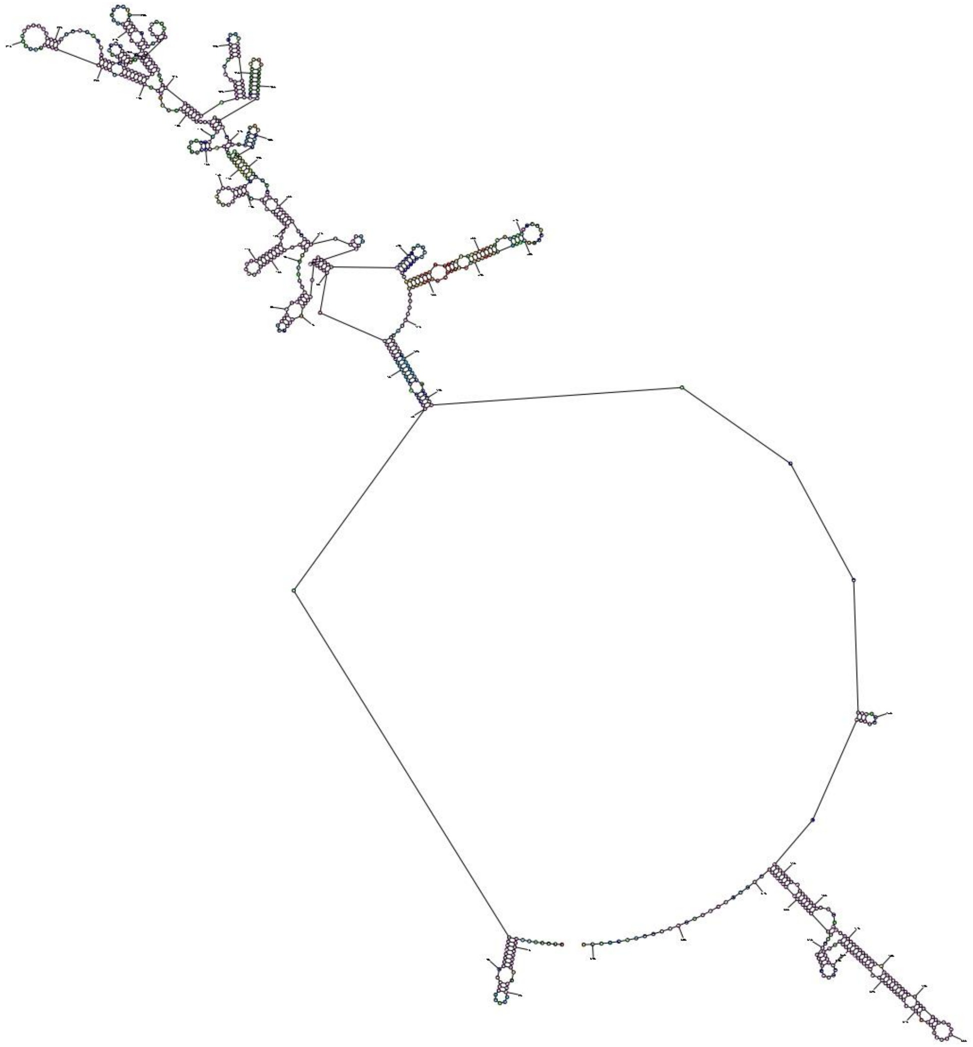

Probability >= 99%
99% > Probability >= 95%
95% > Probability >= 90%
90% > Probability >= 80%
80% > Probability >= 70%
70% > Probability >= 60%
60% > Probability >= 50%
50% > Probability

ENERGY = -77.4 Metopaulias control ...

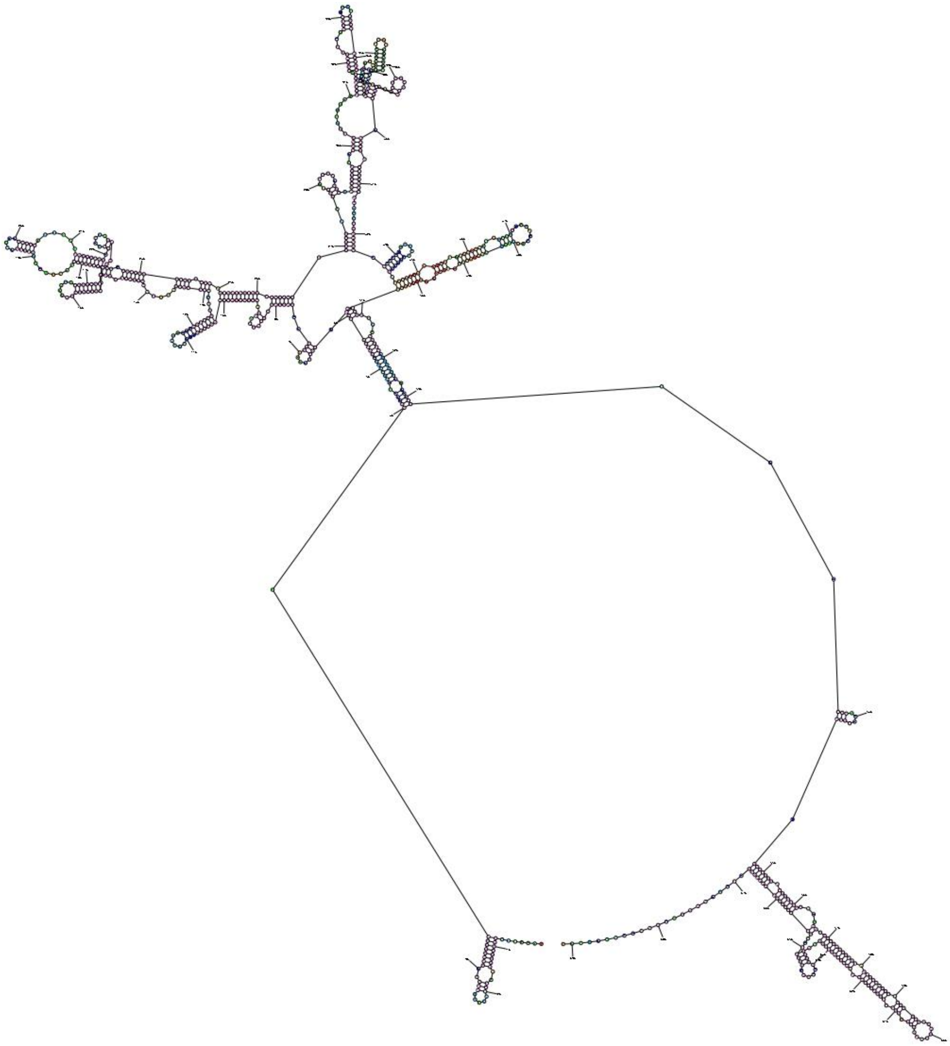

Probability >= 99%
99% > Probability >= 95%
95% > Probability >= 90%
90% > Probability >= 80%
80% > Probability >= 70%
70% > Probability >= 60%
60% > Probability >= 50%
50% > Probability

ENERGY = -77.2 Metopaulias control ...

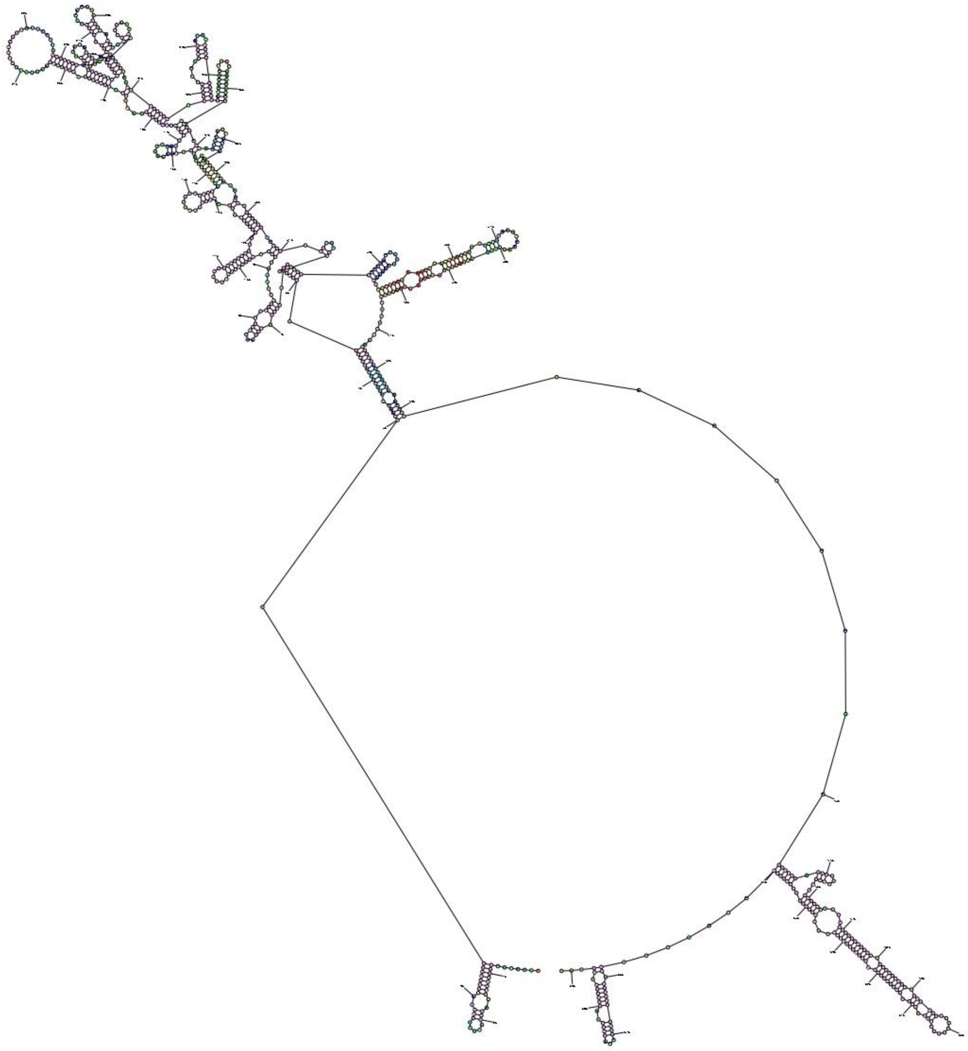

Probability >= 99%
99% > Probability >= 95%
95% > Probability >= 90%
90% > Probability >= 80%
80% > Probability >= 70%
70% > Probability >= 60%
60% > Probability >= 50%
50% > Probability

ENERGY = -77.2 Metopaulias control ...

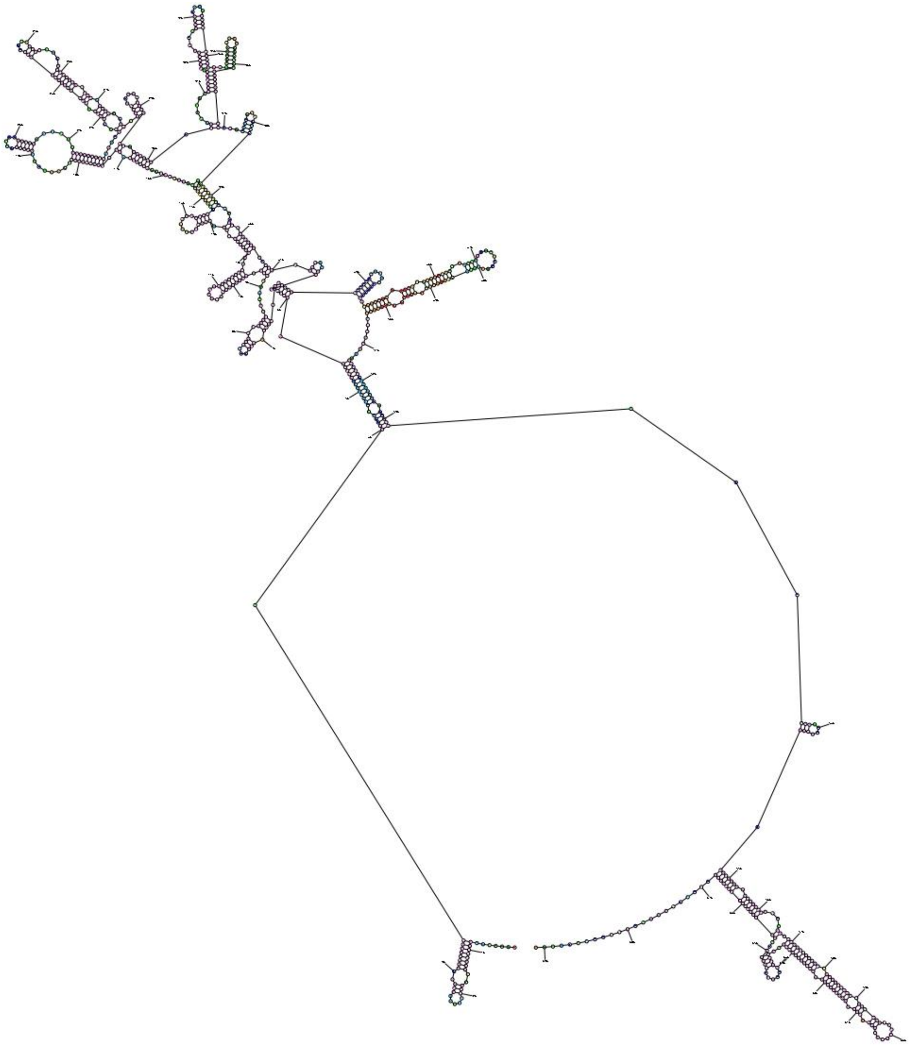

Probability >= 99%
99% > Probability >= 95%
95% > Probability >= 90%
90% > Probability >= 80%
80% > Probability >= 70%
70% > Probability >= 60%
60% > Probability >= 50%
50% > Probability

ENERGY = -77.1 Metopaulias control ...

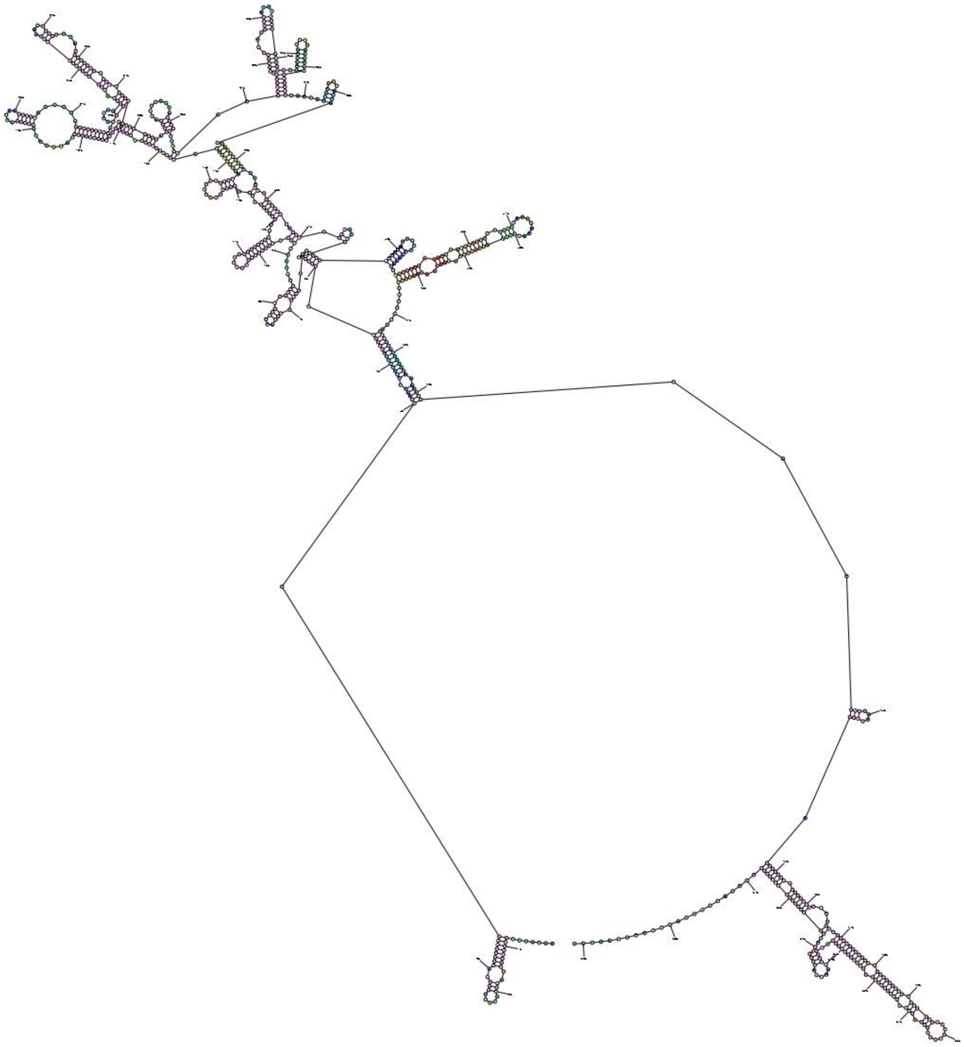

Probability >= 99%
99% > Probability >= 95%
95% > Probability >= 90%
90% > Probability >= 80%
80% > Probability >= 70%
70% > Probability >= 60%
60% > Probability >= 50%
50% > Probability

ENERGY = -77.1 Metopaulias control ...

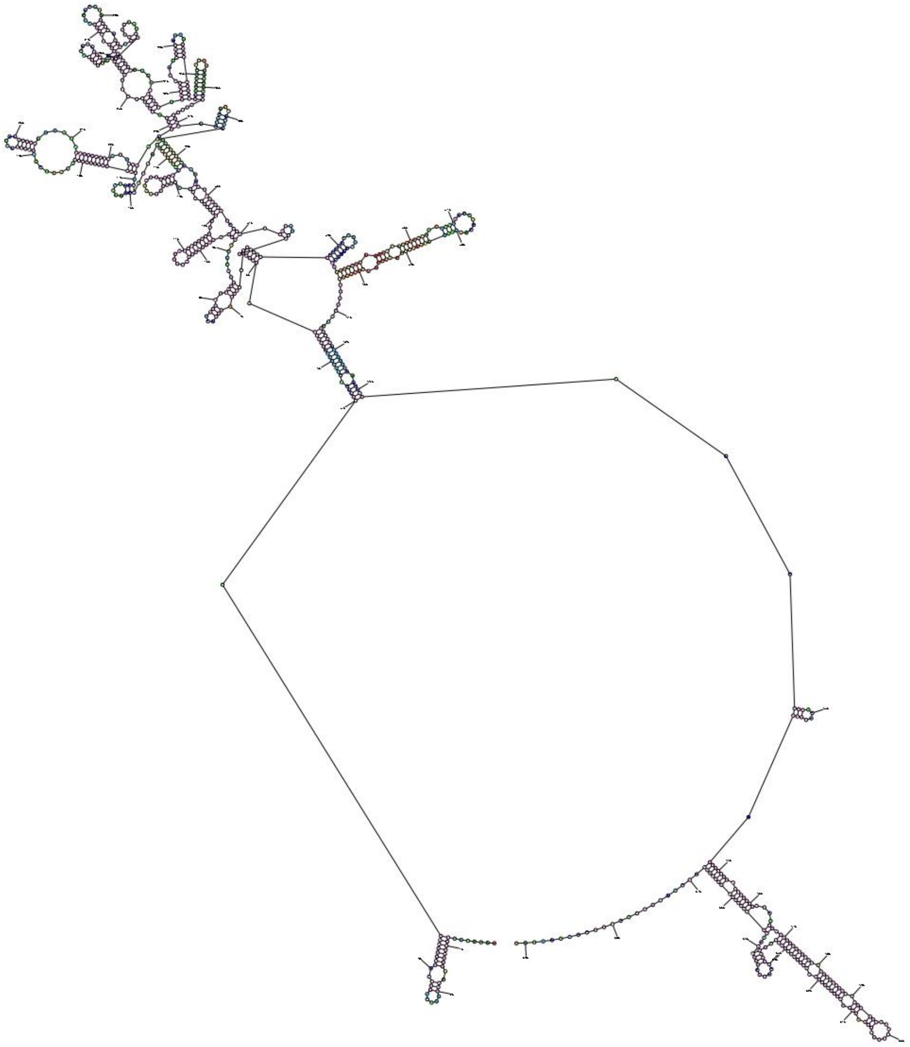

Probability >= 99%
99% > Probability >= 95%
95% > Probability >= 90%
90% > Probability >= 80%
80% > Probability >= 70%
70% > Probability >= 60%
60% > Probability >= 50%
50% > Probability

ENERGY = -77.1 Metopaulias control ...

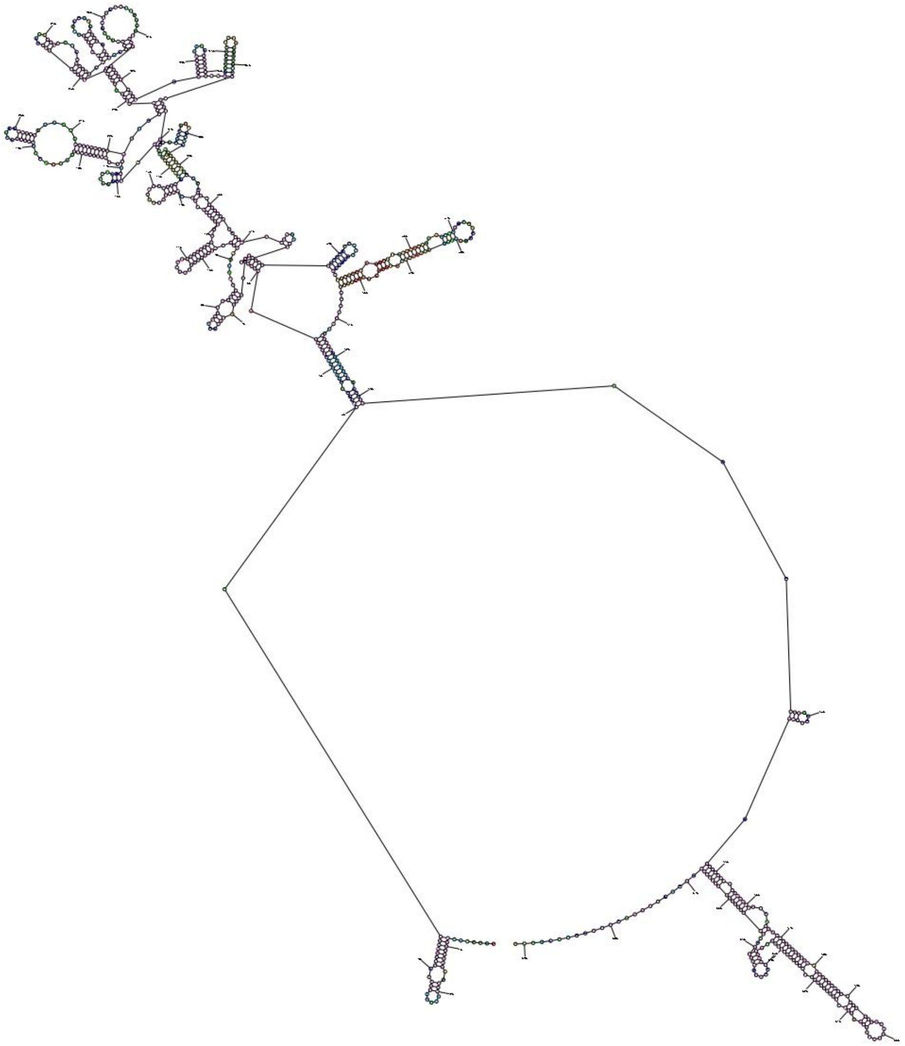

Probability >= 99%
99% > Probability >= 95%
95% > Probability >= 90%
90% > Probability >= 80%
80% > Probability >= 70%
70% > Probability >= 60%
60% > Probability >= 50%
50% > Probability

ENERGY = -77.1 Metopaulias control ...

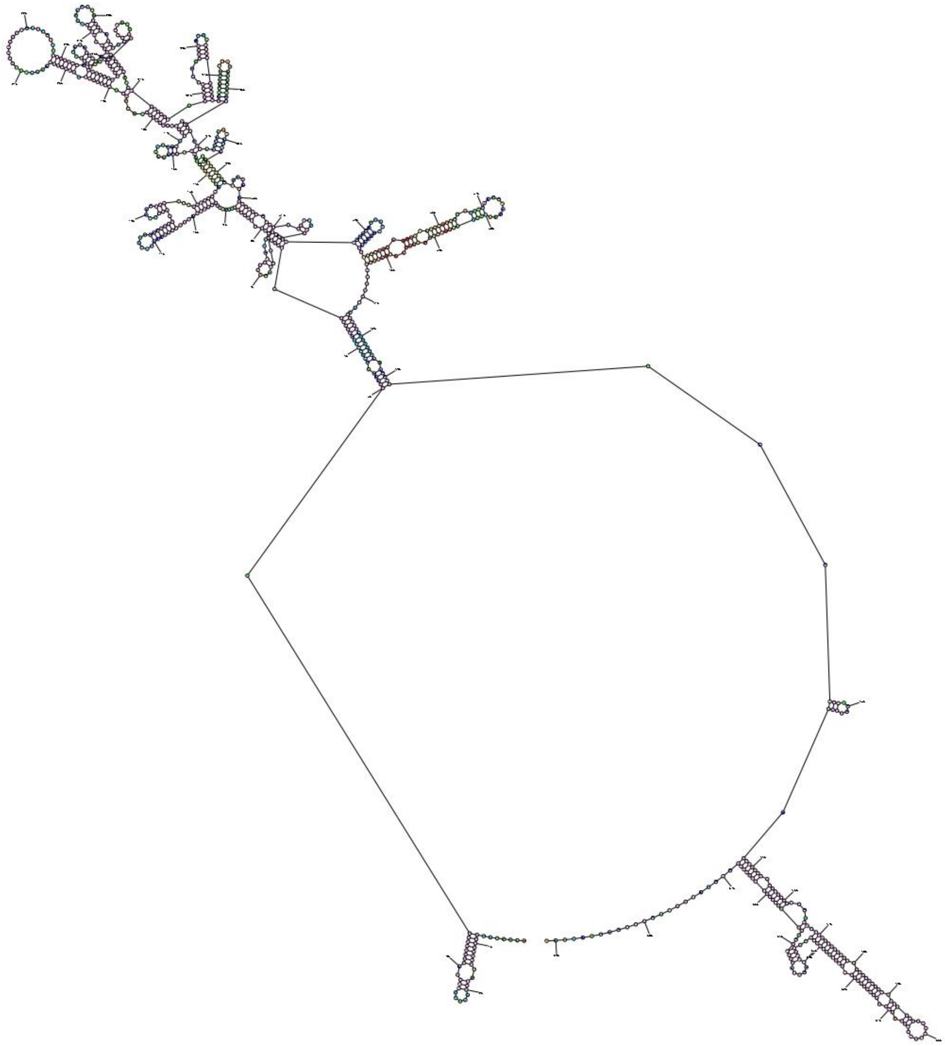

Probability >= 99%
99% > Probability >= 95%
95% > Probability >= 90%
90% > Probability >= 80%
80% > Probability >= 70%
70% > Probability >= 60%
60% > Probability >= 50%
50% > Probability

ENERGY = -77.0 Metopaulias control ...

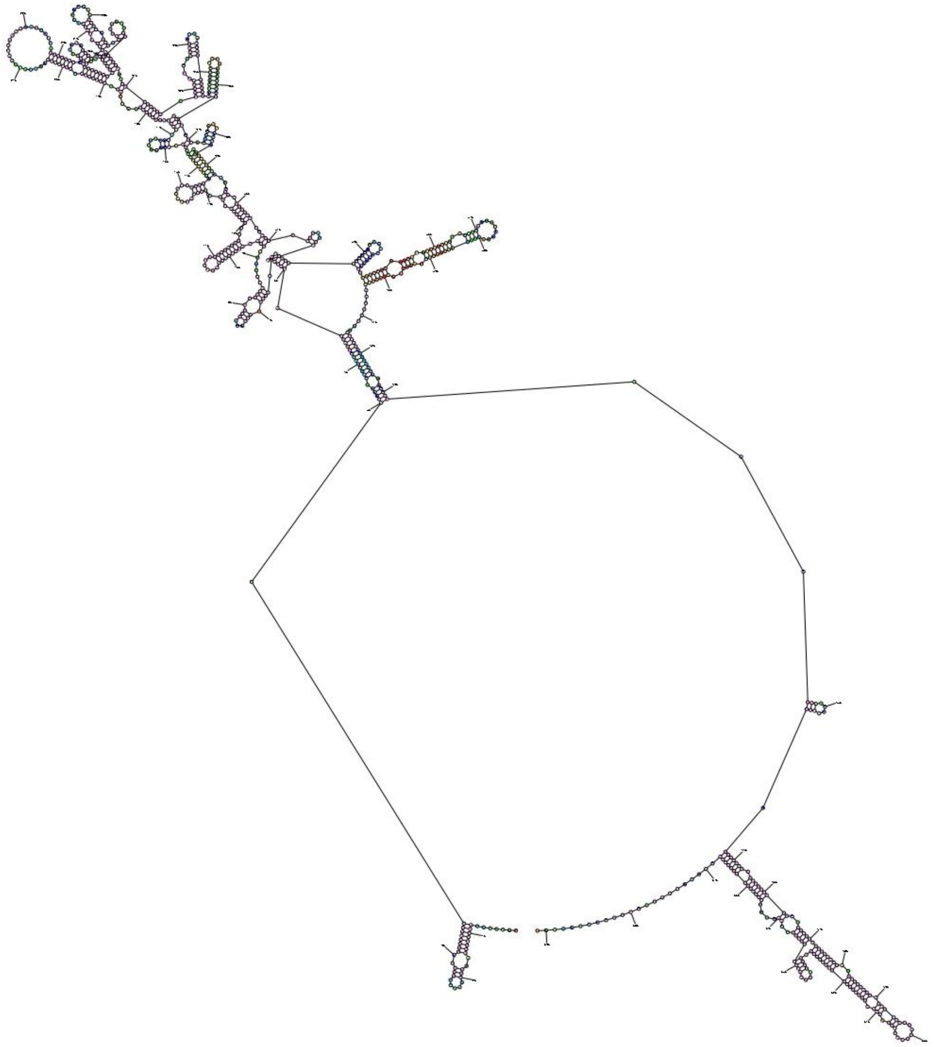

Probability >= 99%
99% > Probability >= 95%
95% > Probability >= 90%
90% > Probability >= 80%
80% > Probability >= 70%
70% > Probability >= 60%
60% > Probability >= 50%
50% > Probability

ENERGY = -77.0 Metopaulias control ...

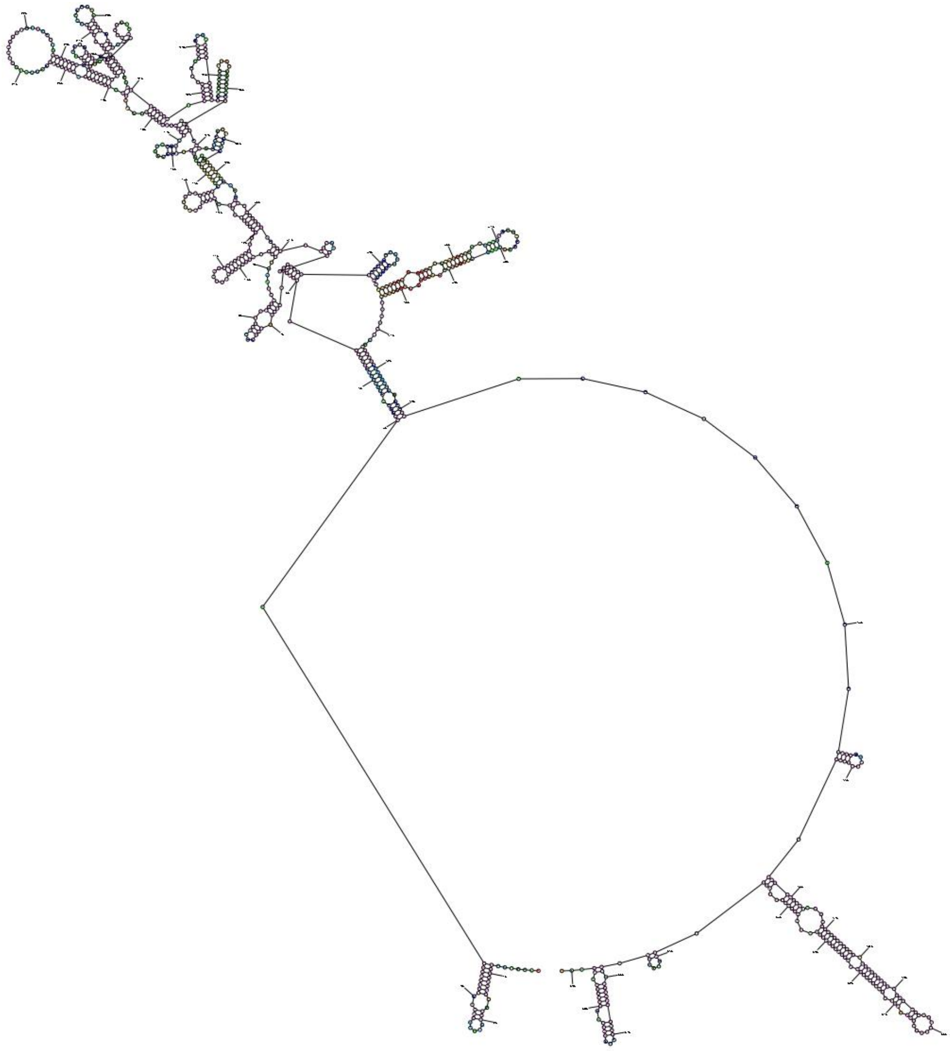

Probability >= 99%
99% > Probability >= 95%
95% > Probability >= 90%
90% > Probability >= 80%
80% > Probability >= 70%
70% > Probability >= 60%
60% > Probability >= 50%
50% > Probability

ENERGY = -76.9 Metopaulias control ...

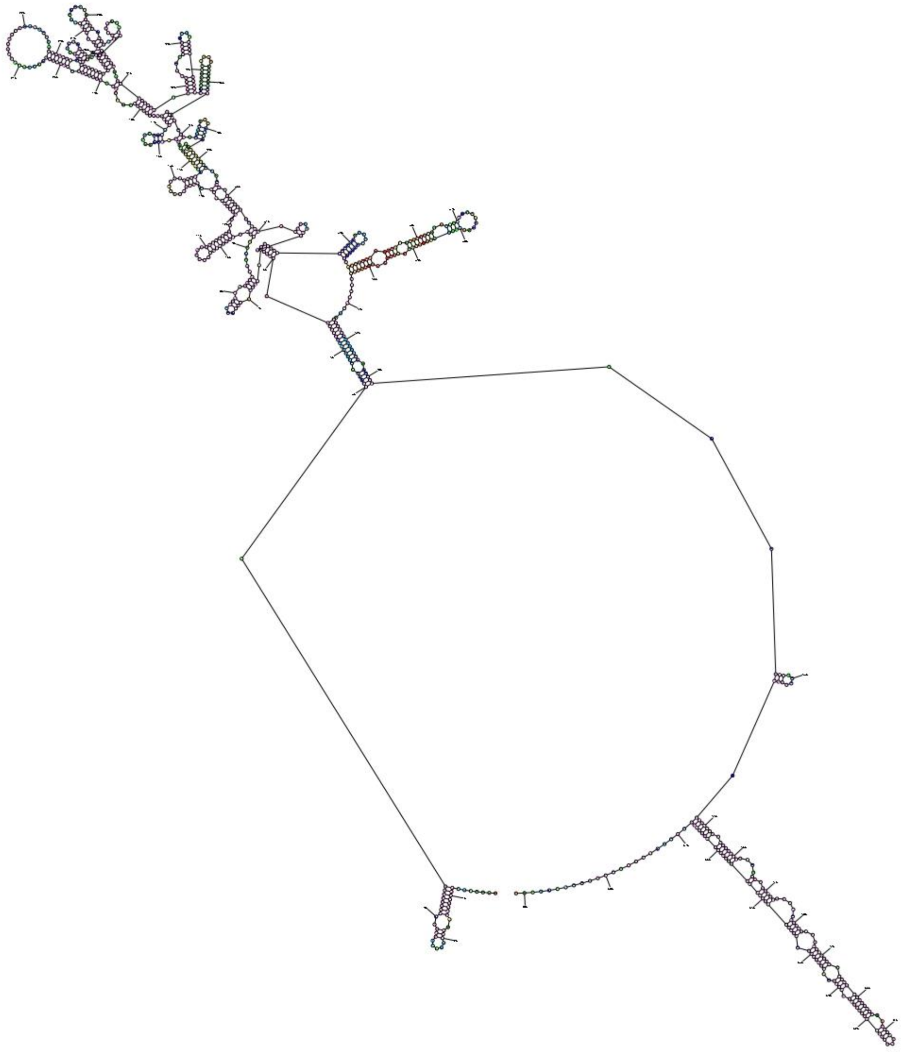

Probability >= 99%
99% > Probability >= 95%
95% > Probability >= 90%
90% > Probability >= 80%
80% > Probability >= 70%
70% > Probability >= 60%
60% > Probability >= 50%
50% > Probability

ENERGY = -76.9 Metopaulias control ...
